# Supplementary material for: The ER membrane protein complex interacts cotranslationally to enable biogenesis of multipass membrane proteins
Source: eLife. 2018 May 29;7:e37018. doi: 10.7554/eLife.37018 (PMC5995541; doi:10.7554/eLife.37018)
Supplement: Figure 3—source data 1. [file elife-37018-fig3-data1.pdf]

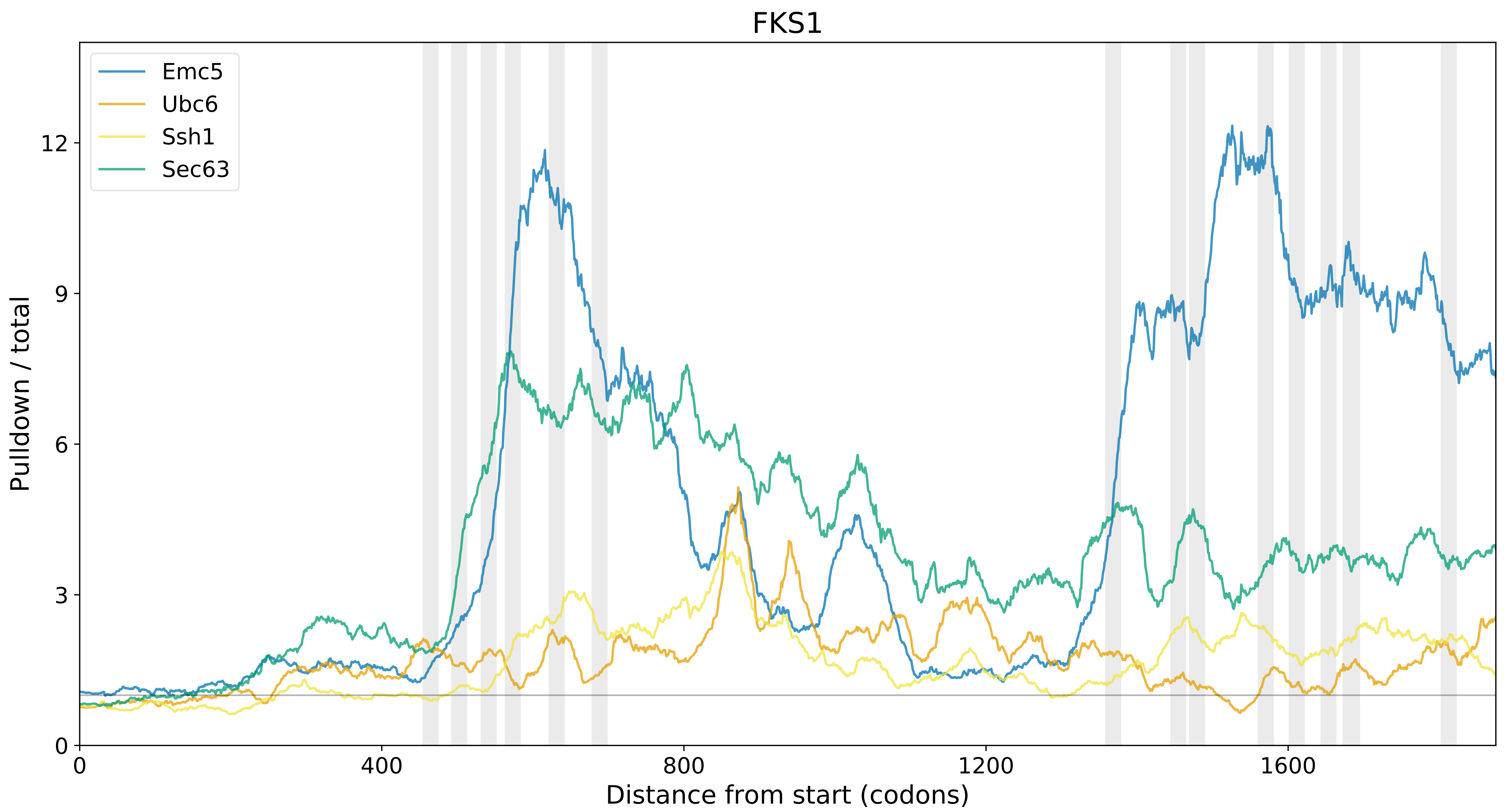
FKS1
Emc5
Ubc6
Ssh1
Sec63
Pulldown / total
0
3
6
9
12
0
400
800
1200
1600
Distance from start (codons)

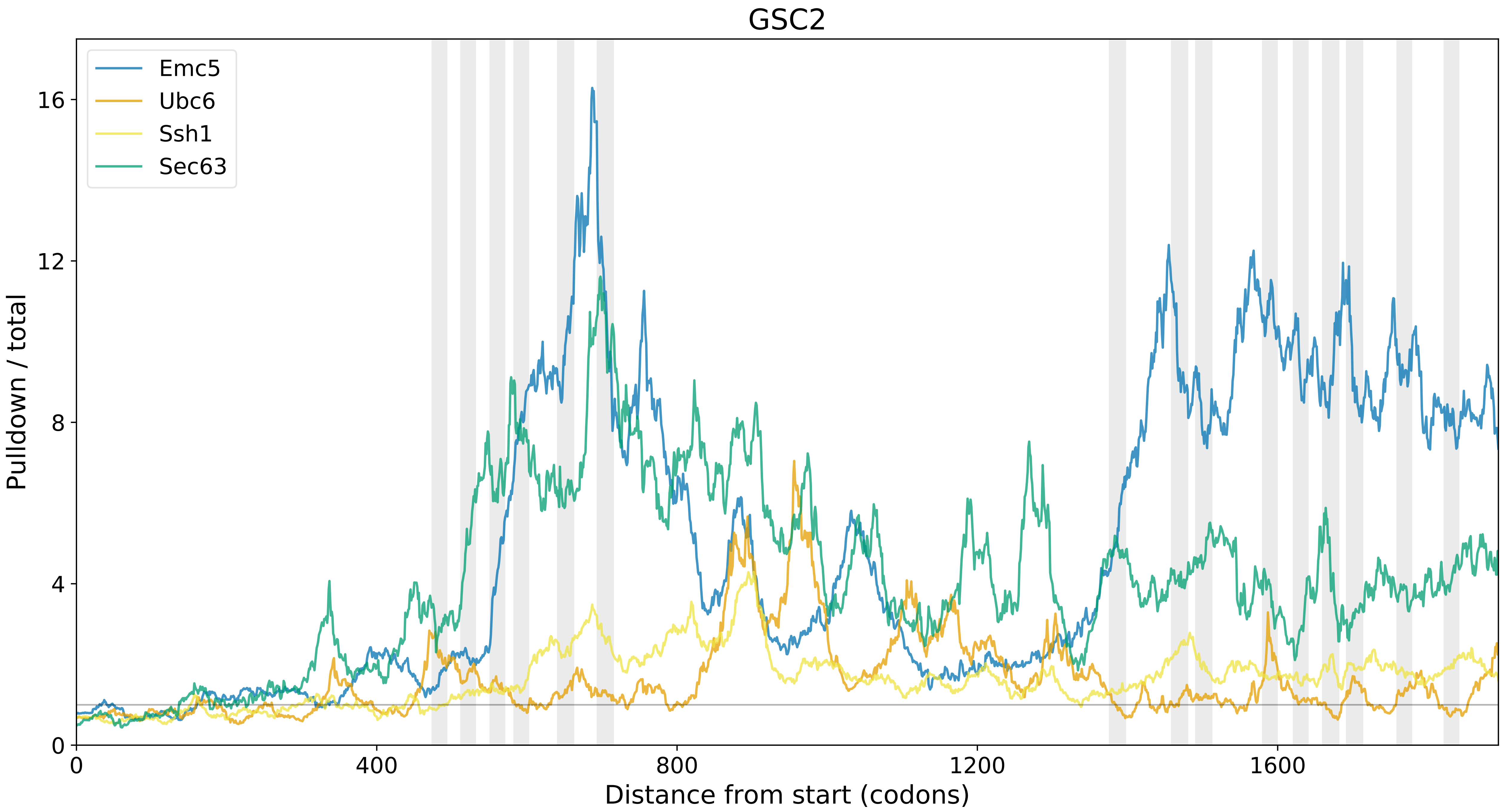

GSC2
Emc5
Ubc6
Ssh1
Sec63
Pulldown / total
Distance from start (codons)
0
4
8
12
16
0
400
800
1200
1600

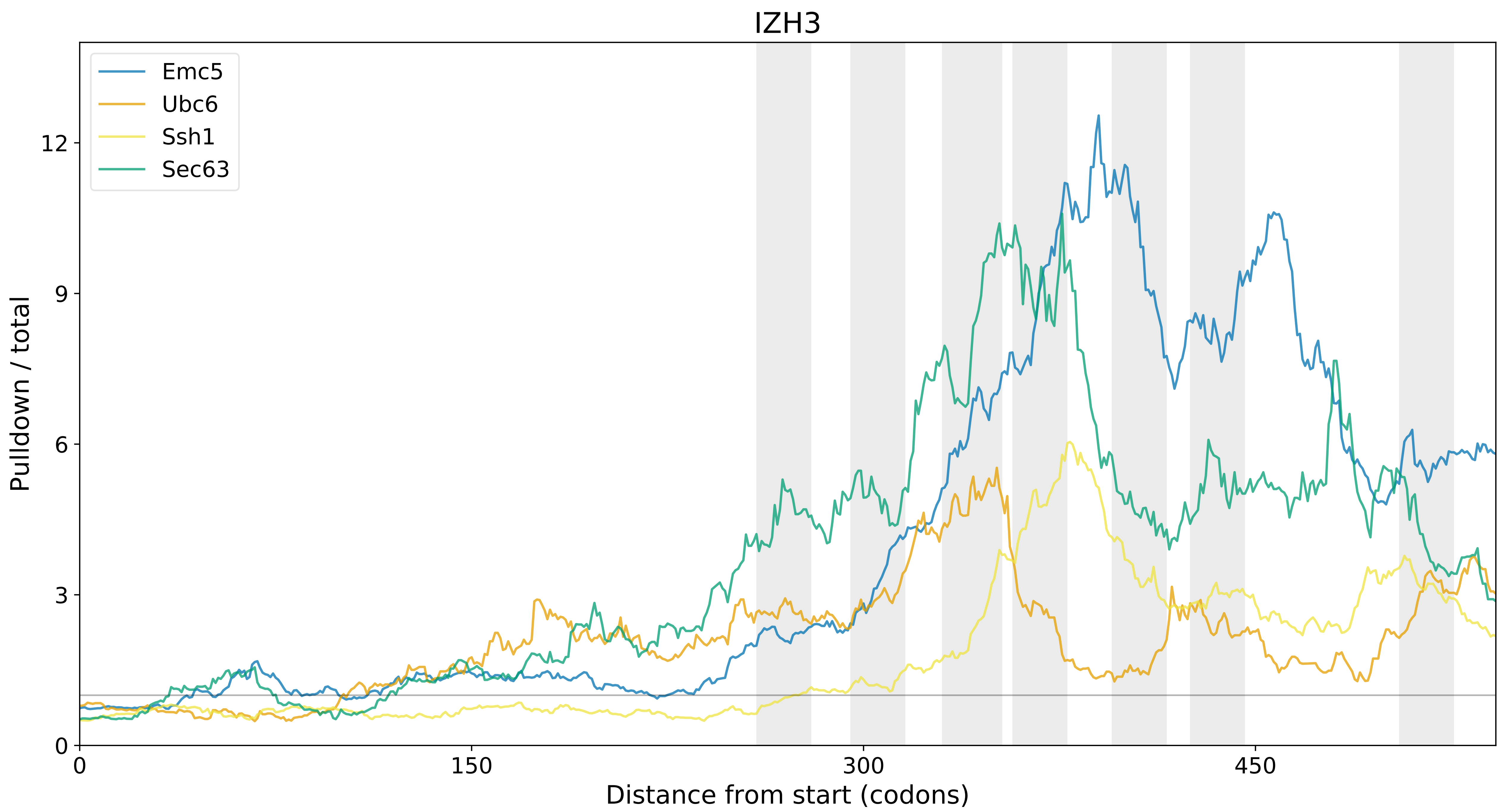

IZH3
Emc5
Ubc6
Ssh1
Sec63
Pulldown / total
Distance from start (codons)
0
3
6
9
12
0
150
300
450

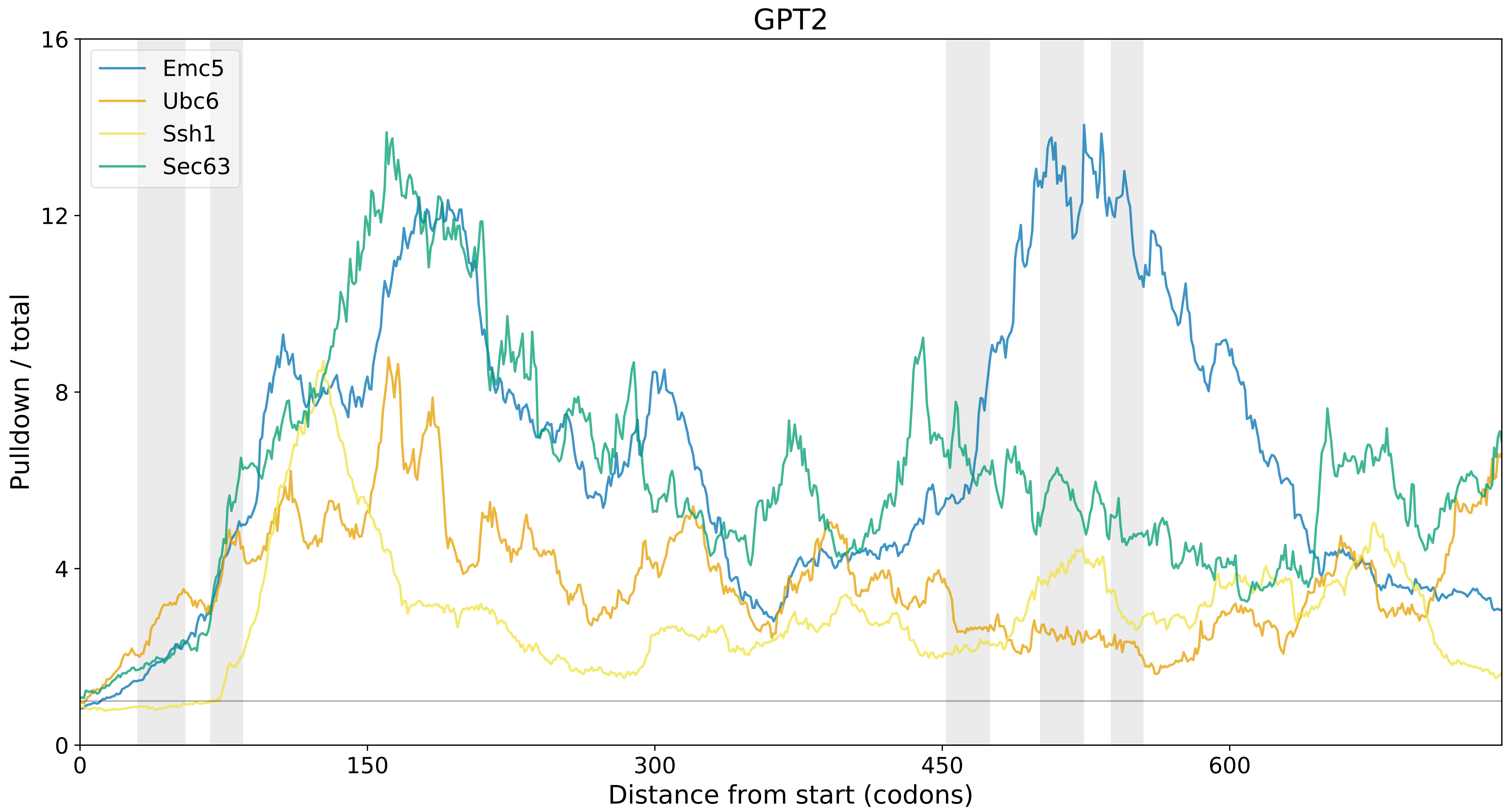

GPT2
Emc5
Ubc6
Ssh1
Sec63
Pulldown / total
Distance from start (codons)
0
4
8
12
16
0
150
300
450
600

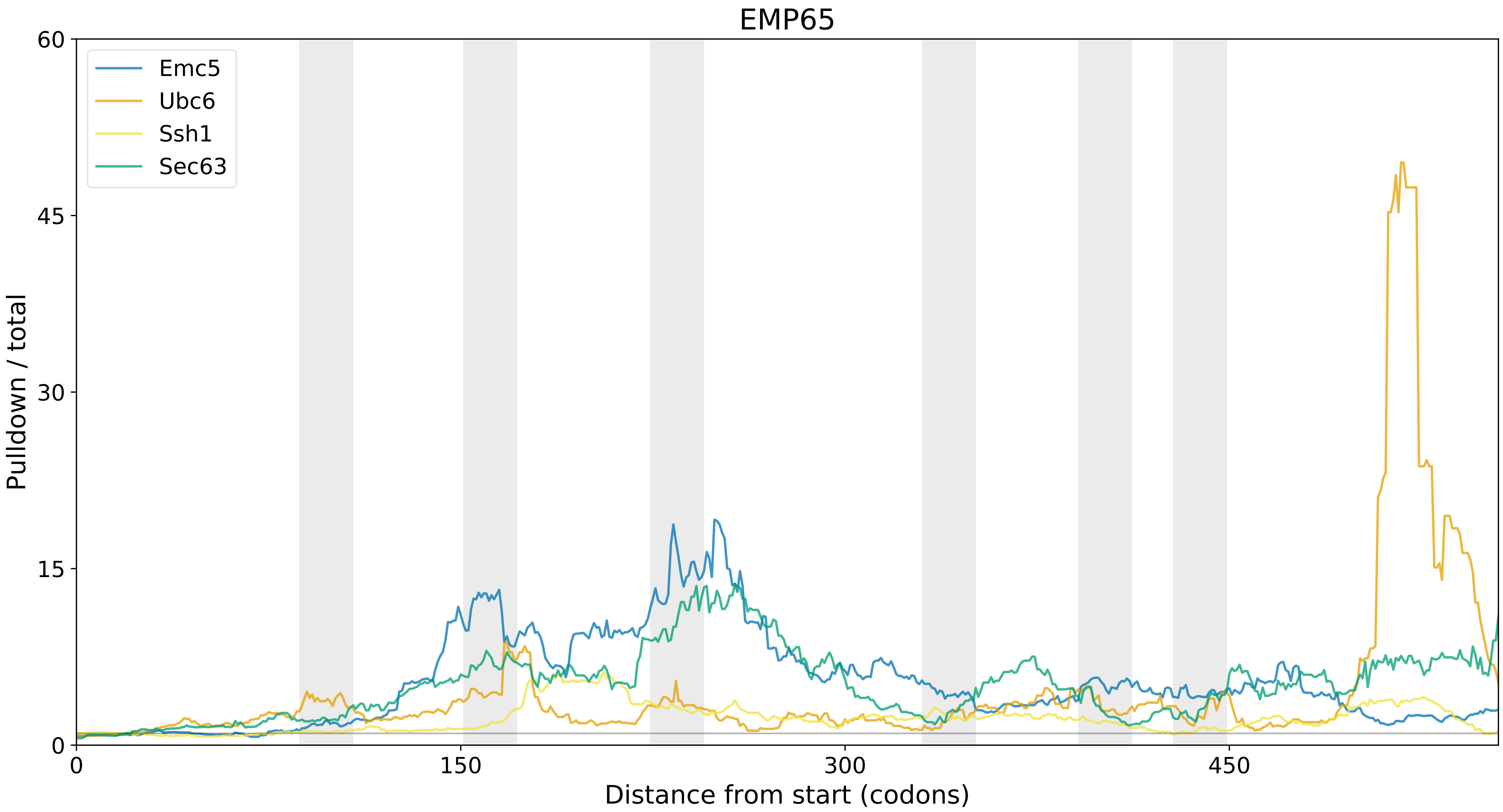

EMP65
Emc5
Ubc6
Ssh1
Sec63
Pulldown / total
Distance from start (codons)
60
45
30
15
0
0
150
300
450

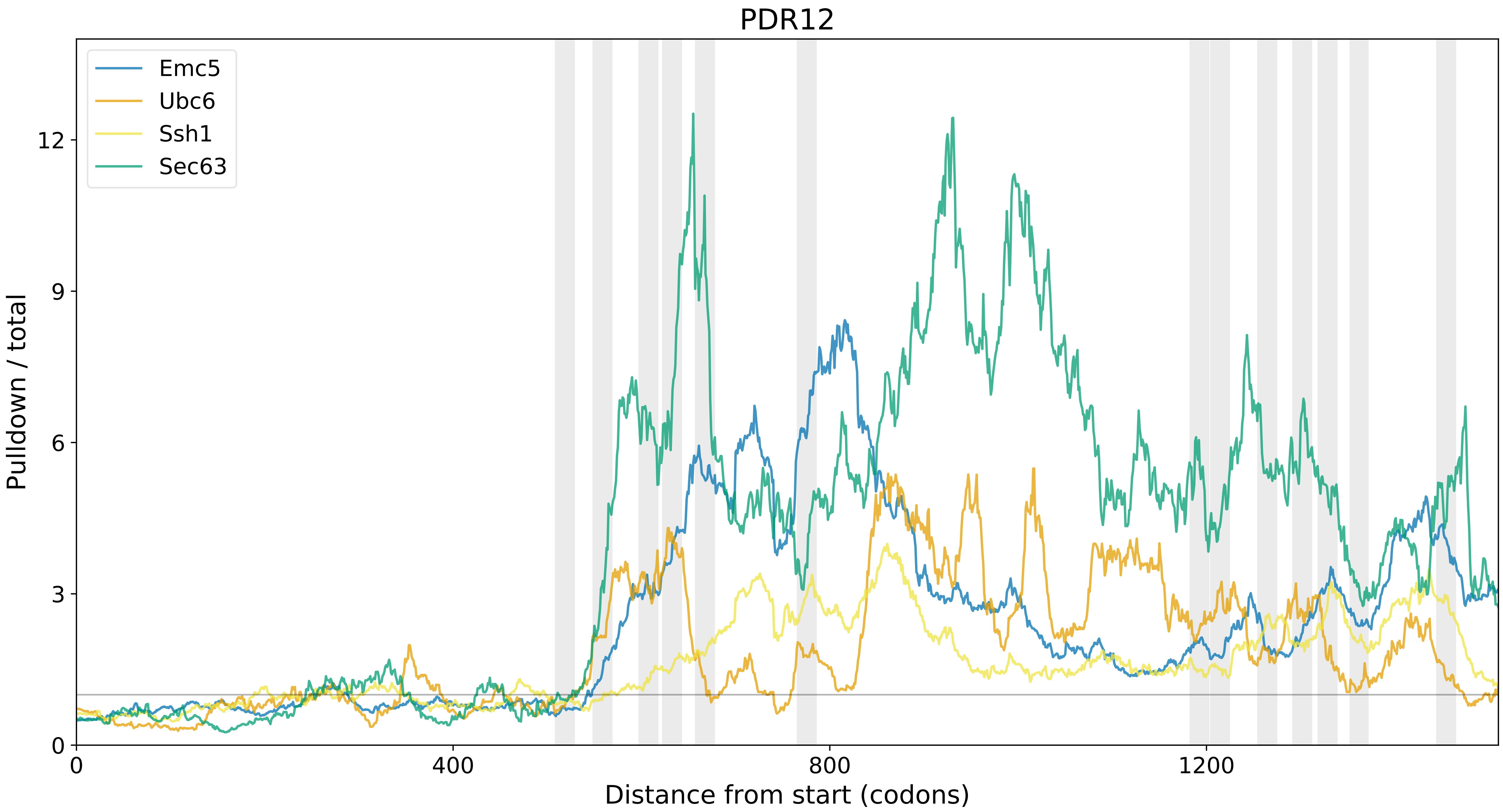

PDR12
Emc5
Ubc6
Ssh1
Sec63
Pulldown / total
0
3
6
9
12
0
400
800
1200
Distance from start (codons)

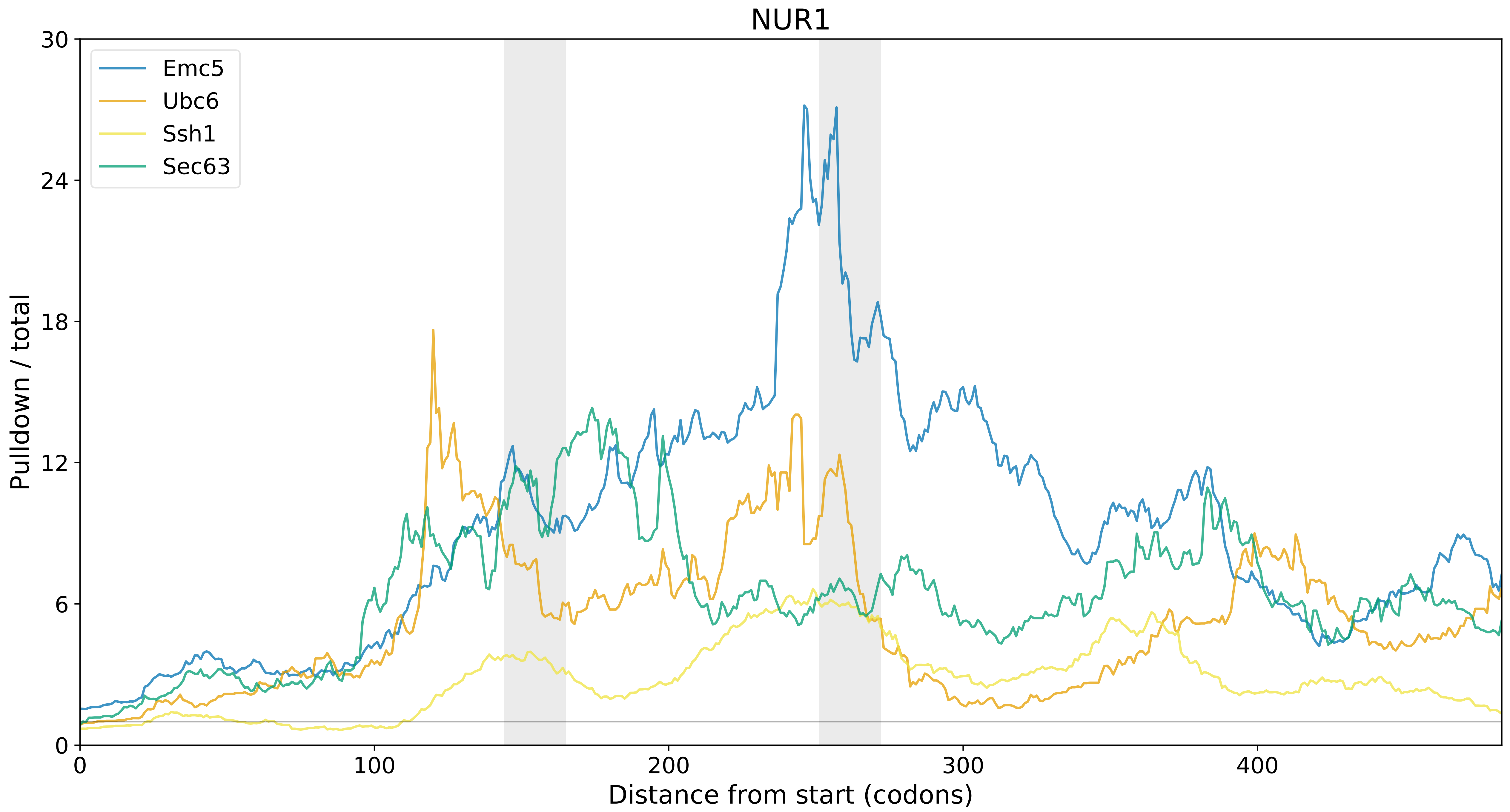

NUR1
Emc5
Ubc6
Ssh1
Sec63
Pulldown / total
Distance from start (codons)
0
6
12
18
24
30
0
100
200
300
400

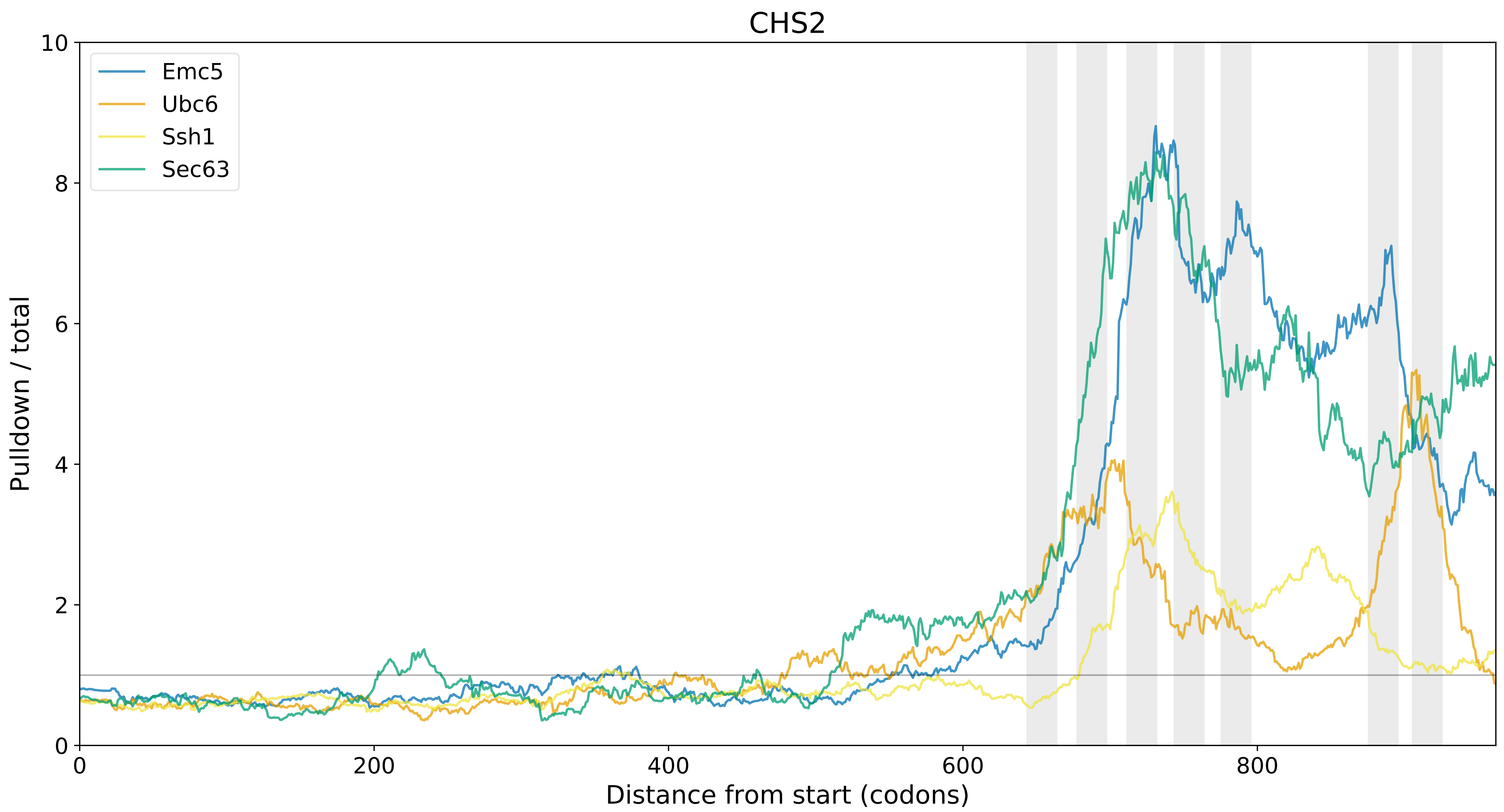

CHS2
Emc5
Ubc6
Ssh1
Sec63
Pulldown / total
Distance from start (codons)
0
2
4
6
8
10
0
200
400
600
800

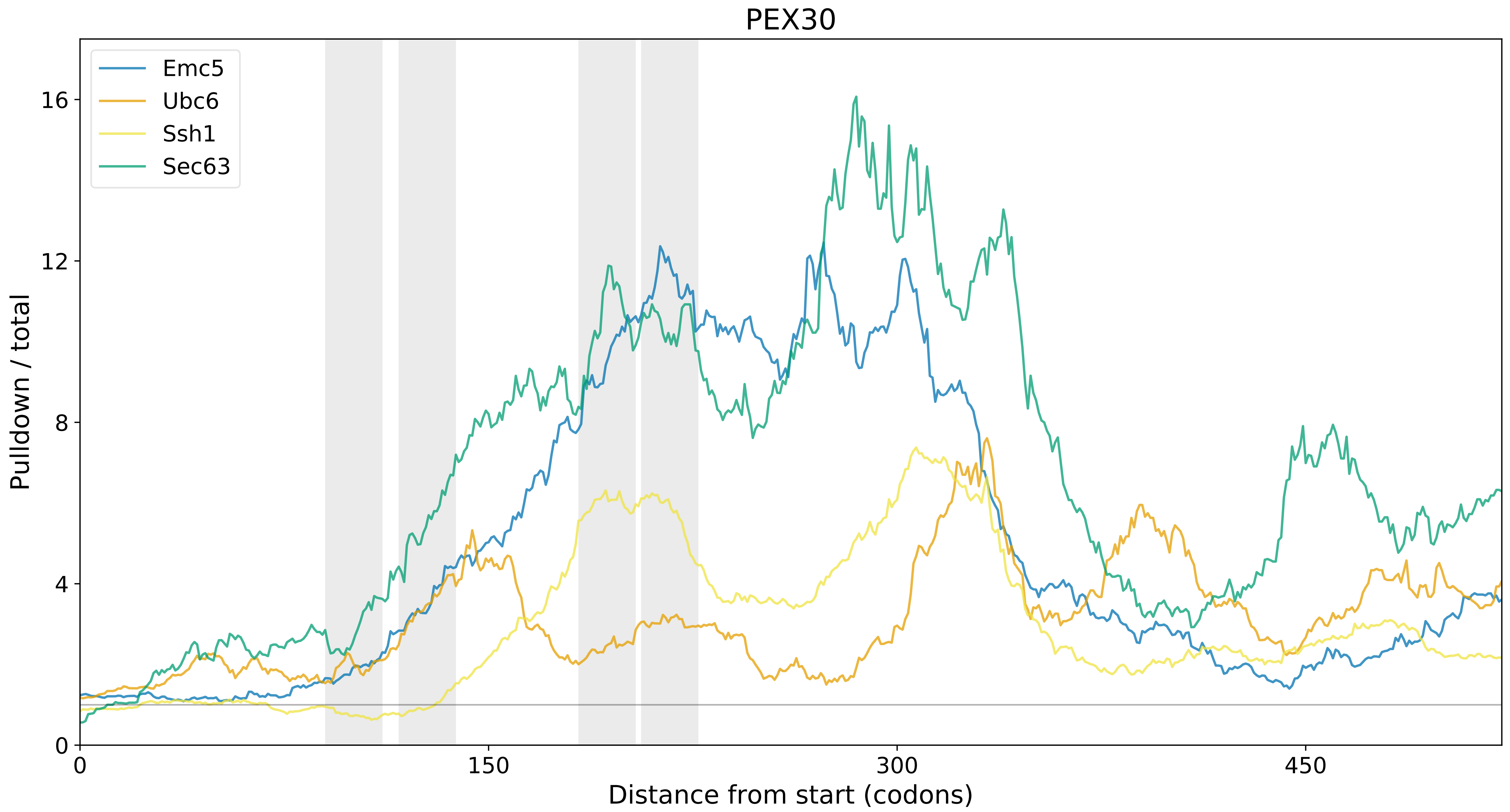

PEX30
Emc5
Ubc6
Ssh1
Sec63
Pulldown / total
Distance from start (codons)
0
4
8
12
16
0
150
300
450

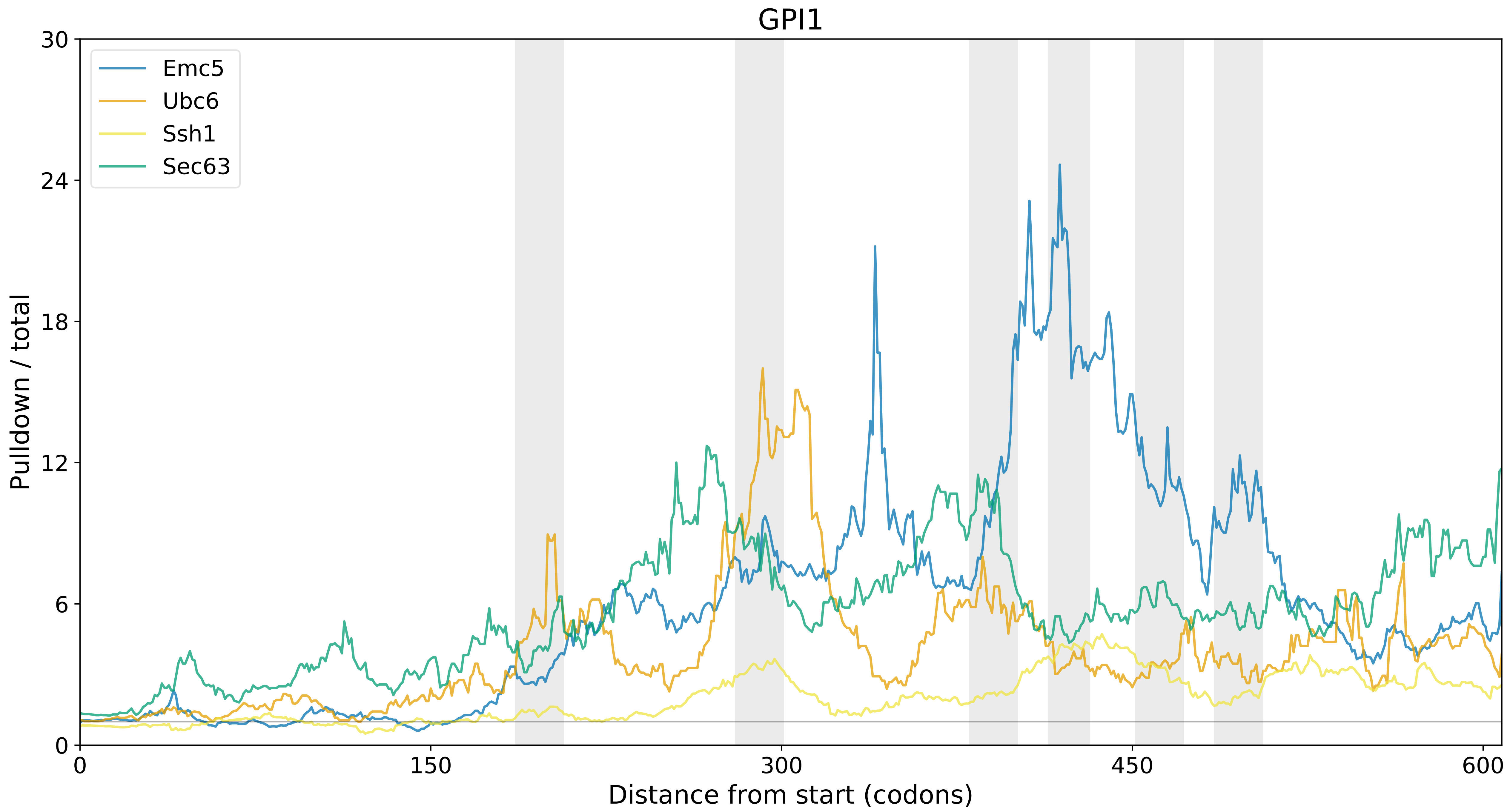

GPI1
Emc5
Ubc6
Ssh1
Sec63
Pulldown / total
0
6
12
18
24
30
0
150
300
450
600
Distance from start (codons)

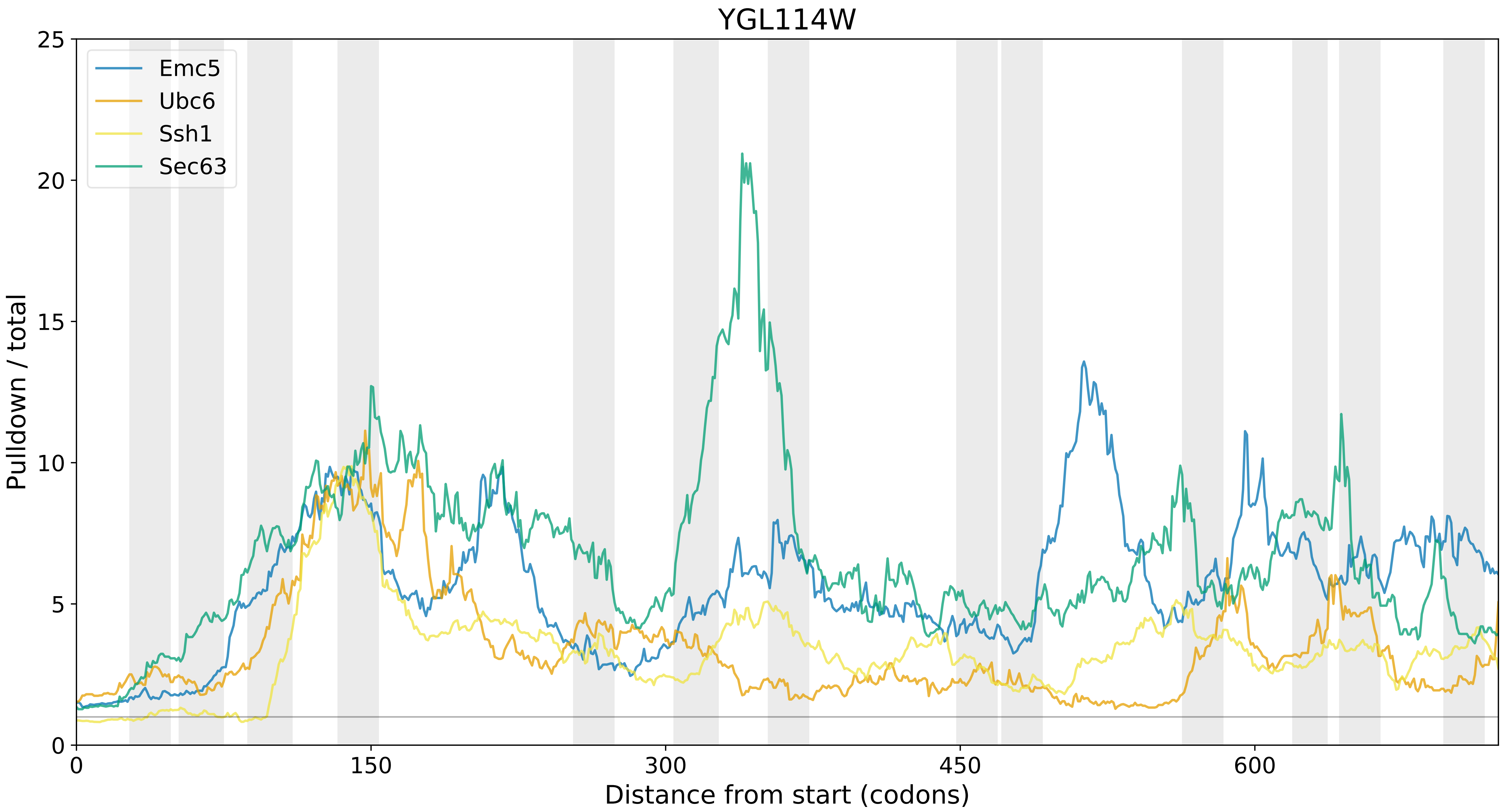
YGL114W
Emc5
Ubc6
Ssh1
Sec63
Pulldown / total
Distance from start (codons)
0
5
10
15
20
25
0
150
300
450
600

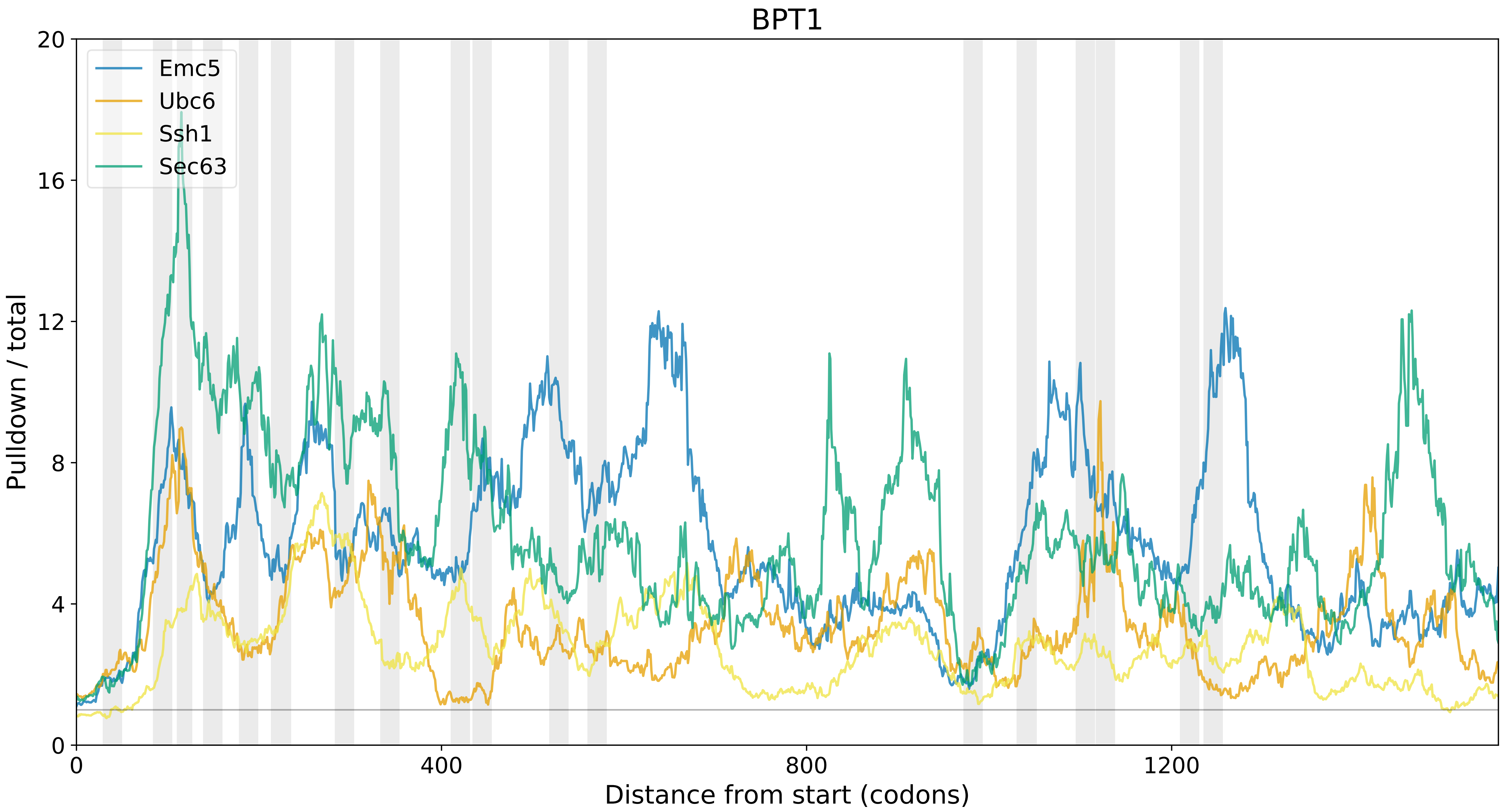

BPT1
Emc5
Ubc6
Ssh1
Sec63
Pulldown / total
Distance from start (codons)
0
4
8
12
16
20
0
400
800
1200

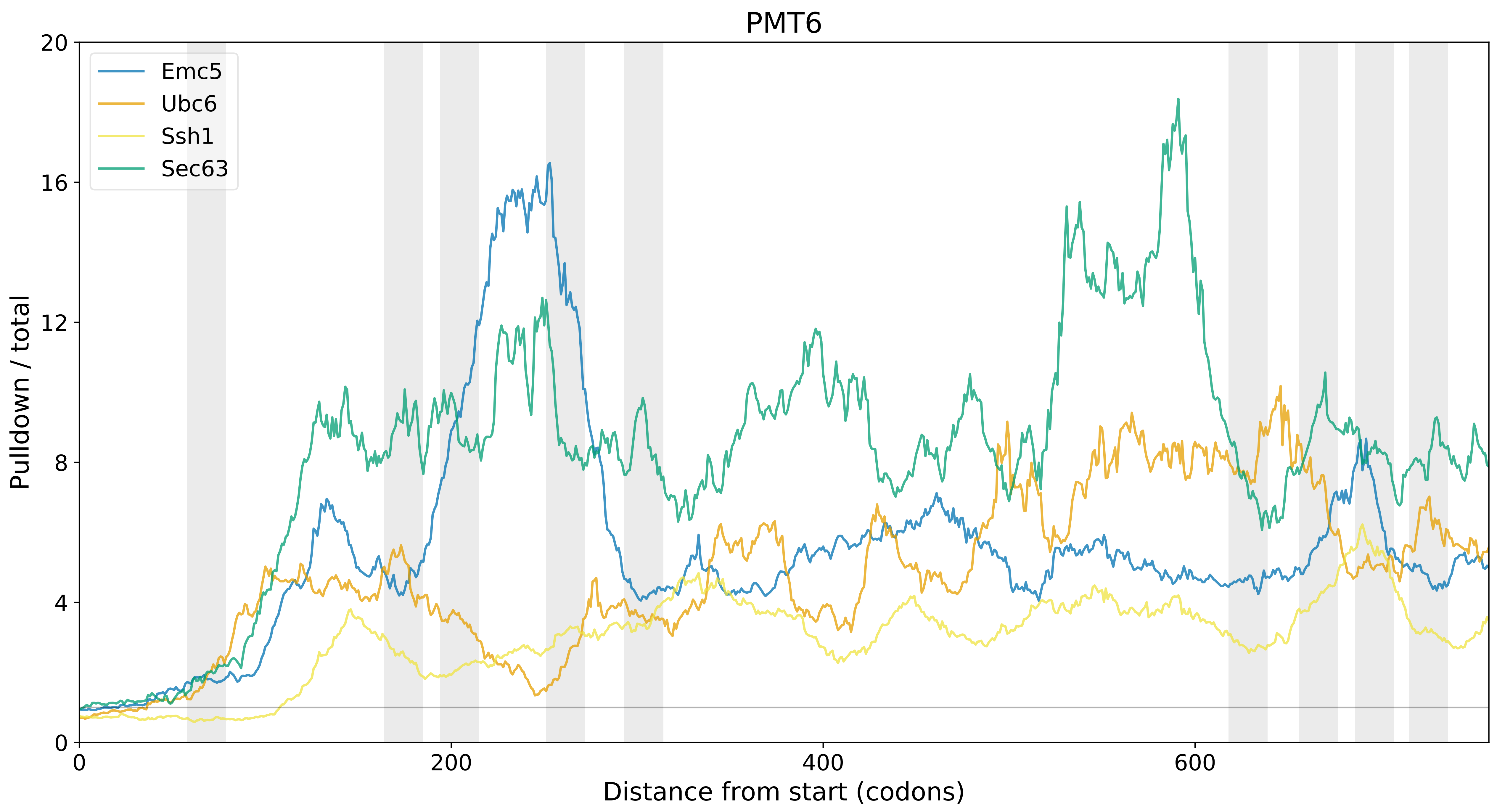

PMT6
Emc5
Ubc6
Ssh1
Sec63
Pulldown / total
Distance from start (codons)
0
4
8
12
16
20
0
200
400
600

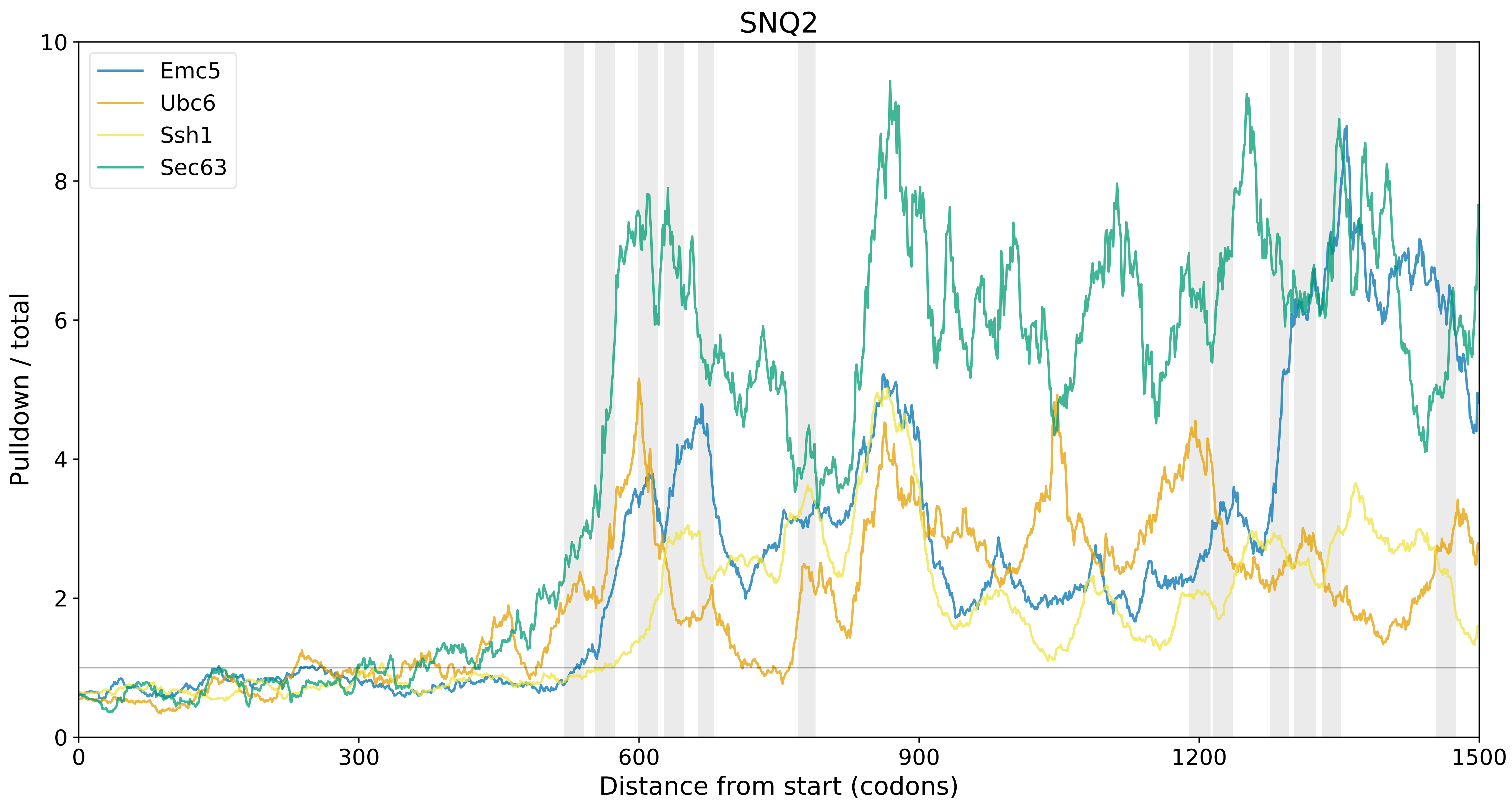
SNQ2
Emc5
Ubc6
Ssh1
Sec63
Pulldown / total
Distance from start (codons)
0
2
4
6
8
10
0
300
600
900
1200
1500

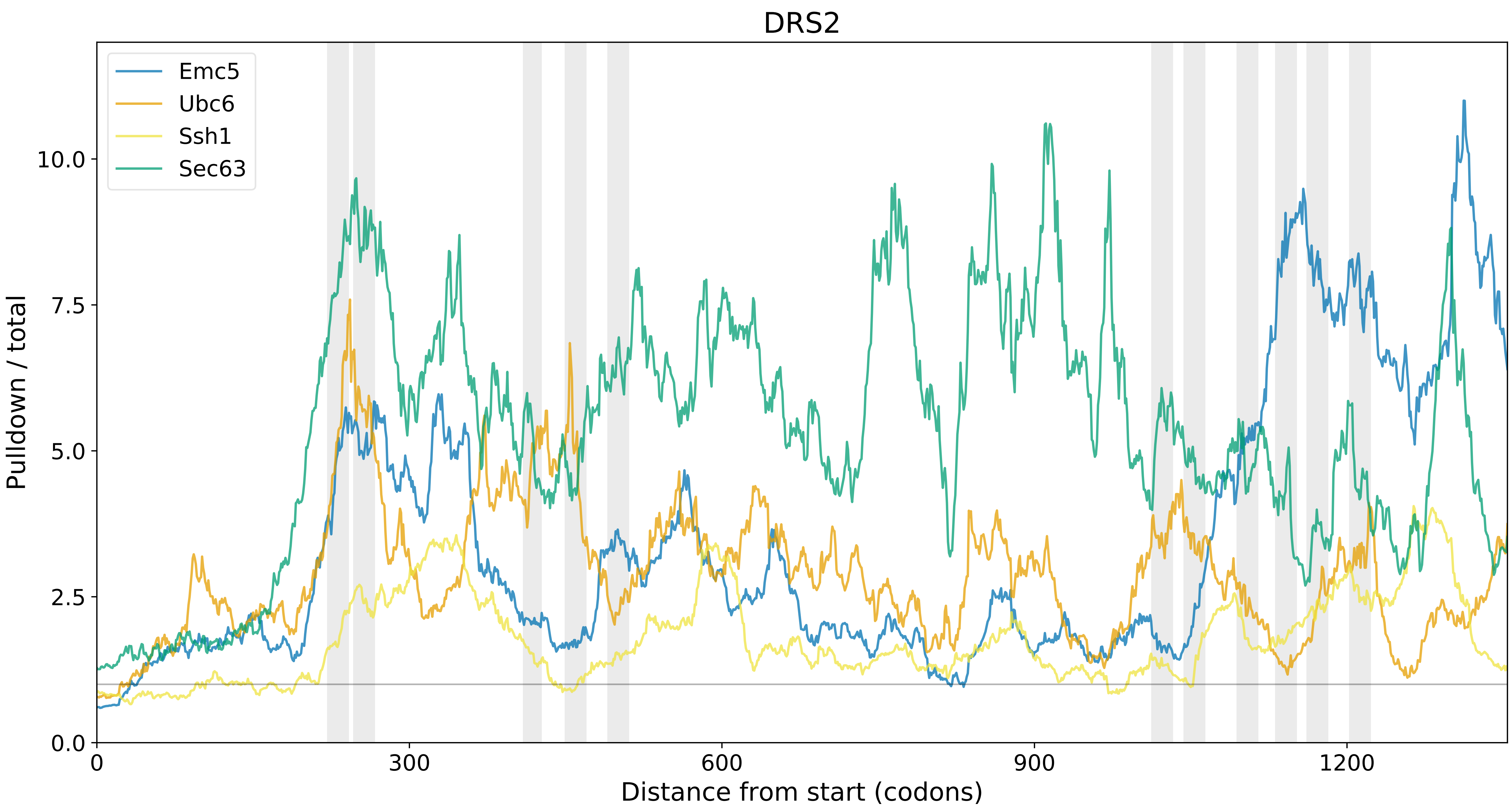

DRS2
Emc5
Ubc6
Ssh1
Sec63
Pulldown / total
Distance from start (codons)
0.0
2.5
5.0
7.5
10.0
0
300
600
900
1200

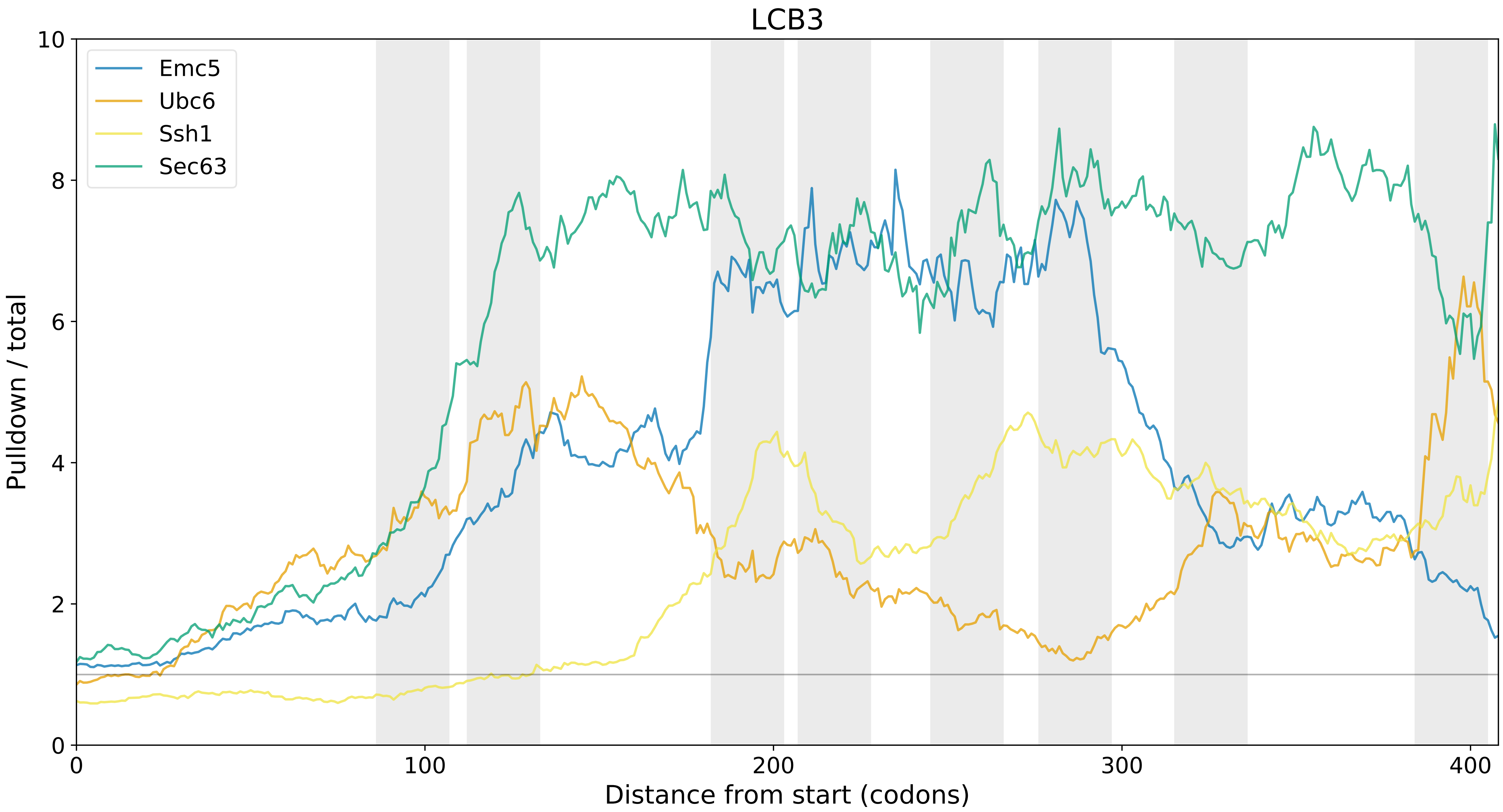

LCB3
Emc5
Ubc6
Ssh1
Sec63
Pulldown / total
Distance from start (codons)
0
2
4
6
8
10
0
100
200
300
400

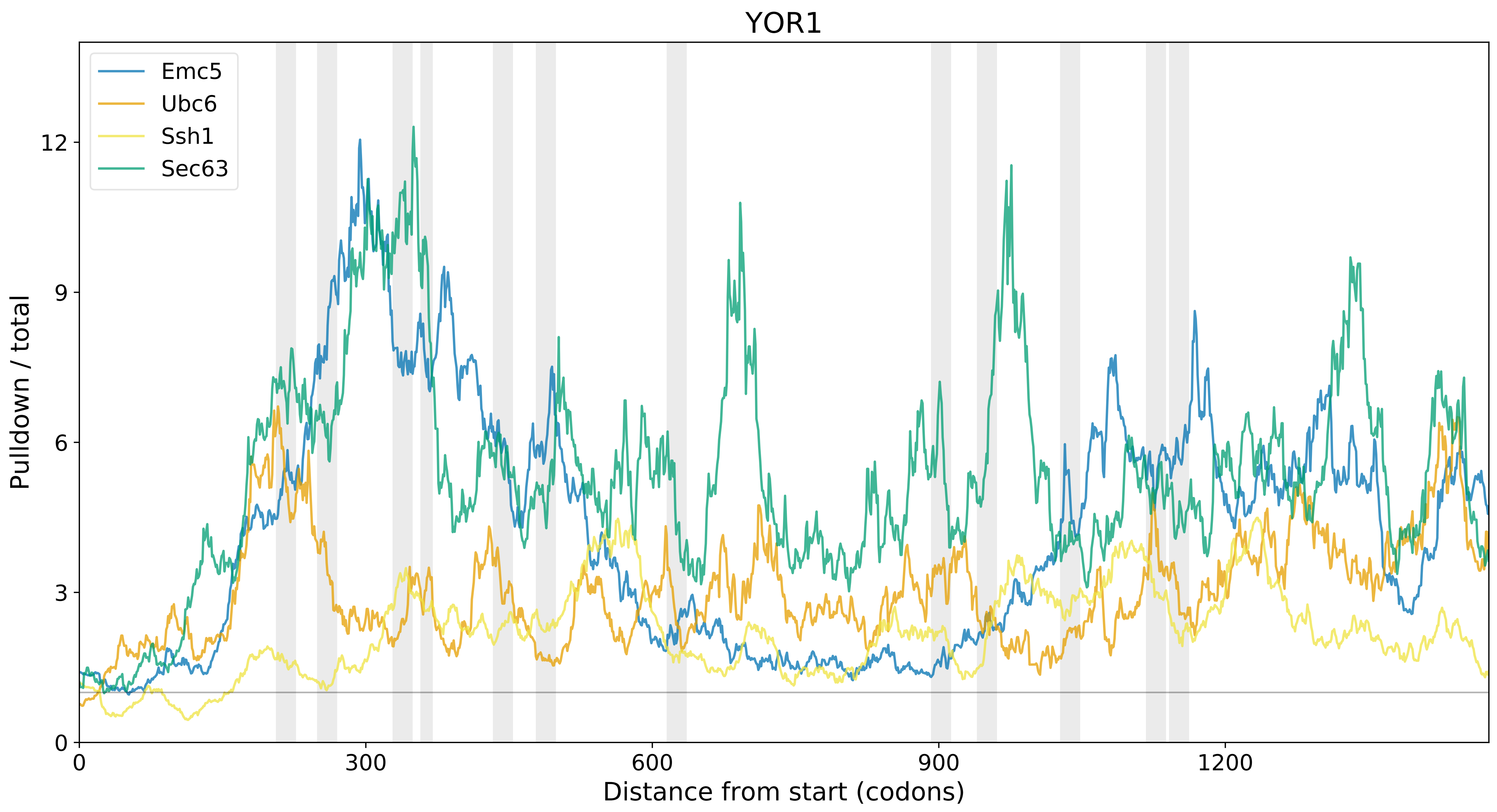

YOR1
Emc5
Ubc6
Ssh1
Sec63
Pulldown / total
Distance from start (codons)
0
3
6
9
12
0
300
600
900
1200

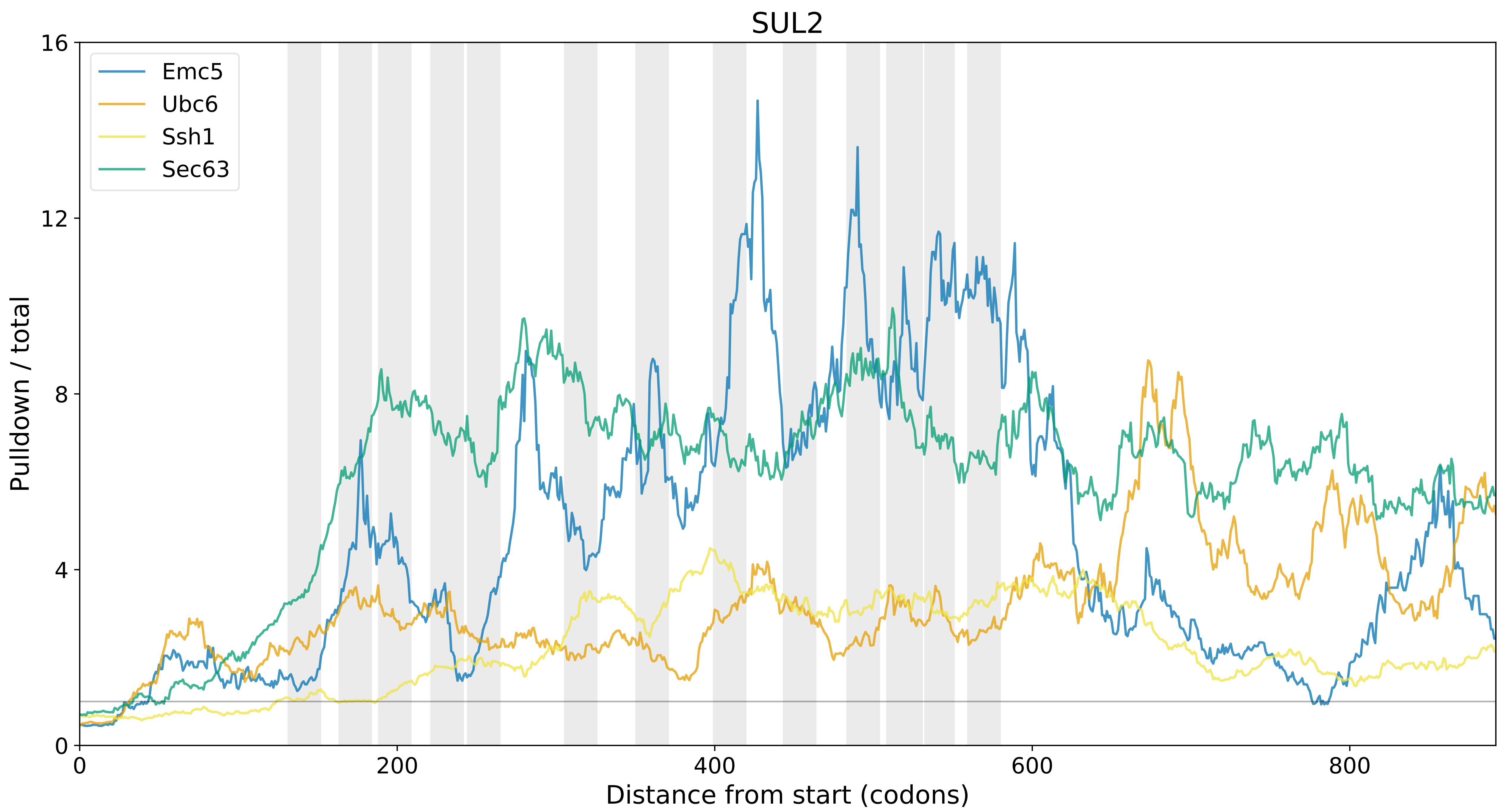
SUL2
Emc5
Ubc6
Ssh1
Sec63
Pulldown / total
Distance from start (codons)
0
4
8
12
16
0
200
400
600
800

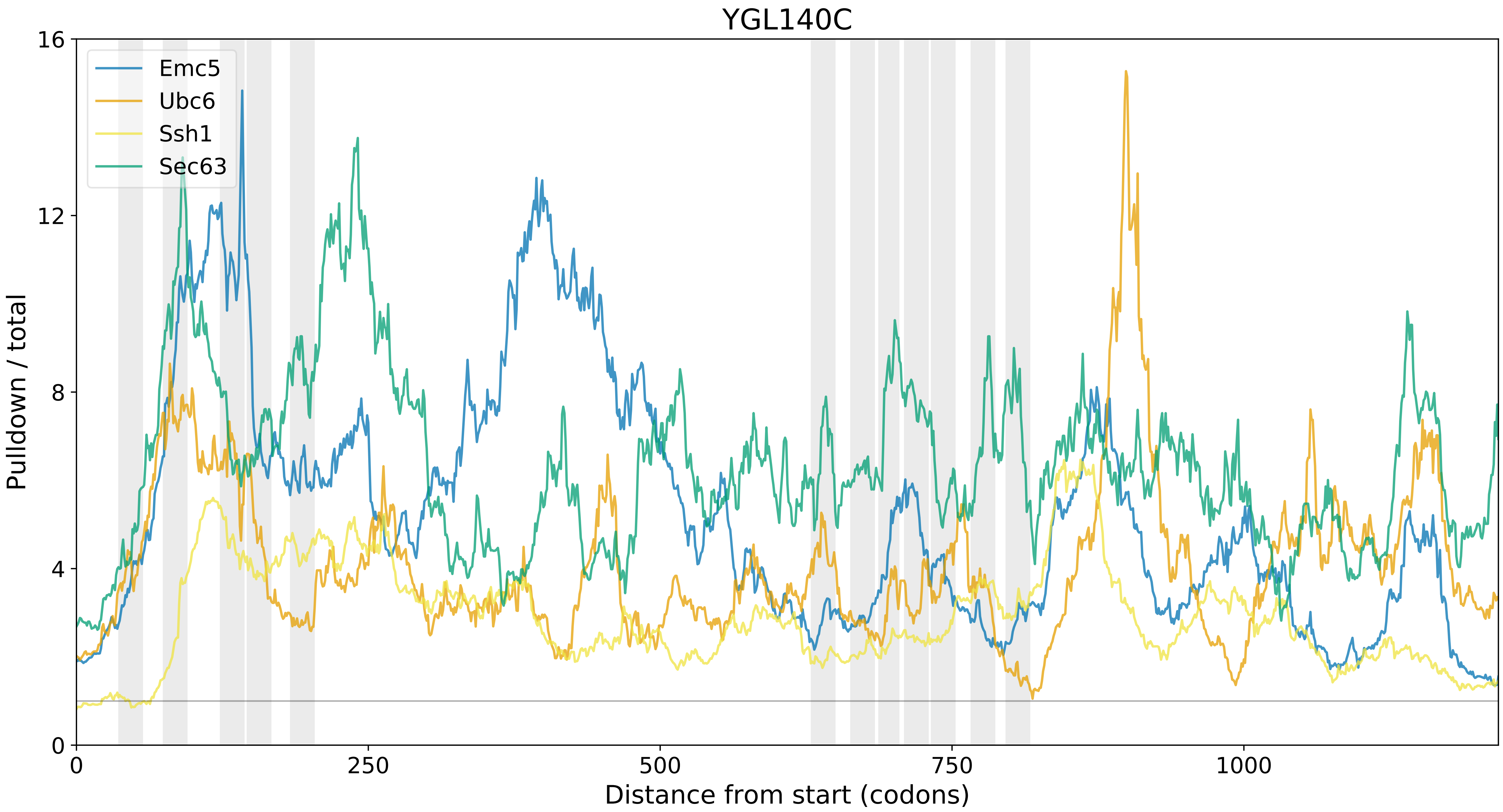

YGL140C
Emc5
Ubc6
Ssh1
Sec63
Pulldown / total
Distance from start (codons)
0
4
8
12
16
0
250
500
750
1000

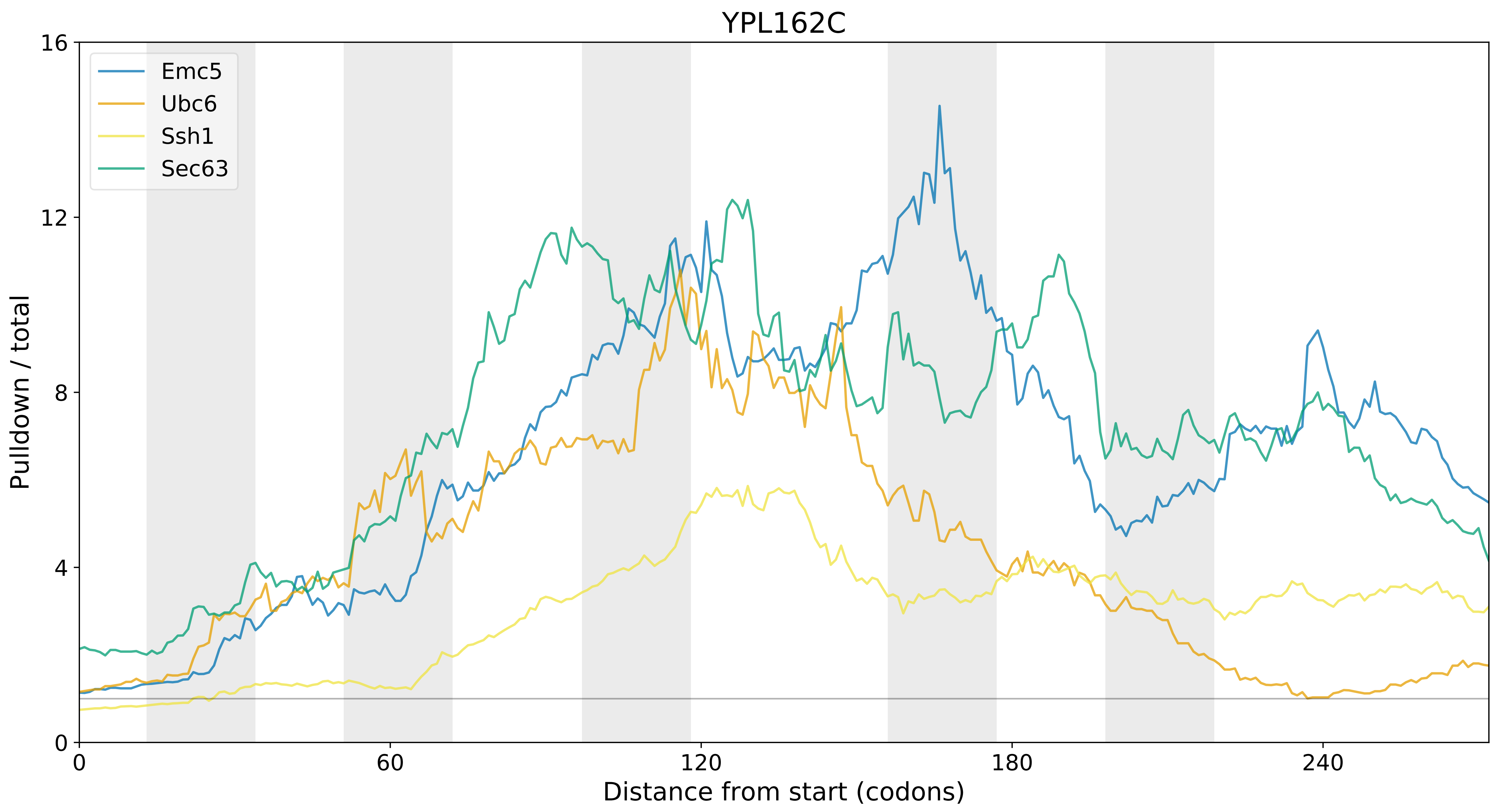
YPL162C
Emc5
Ubc6
Ssh1
Sec63
Pulldown / total
Distance from start (codons)
0
4
8
12
16
0
60
120
180
240

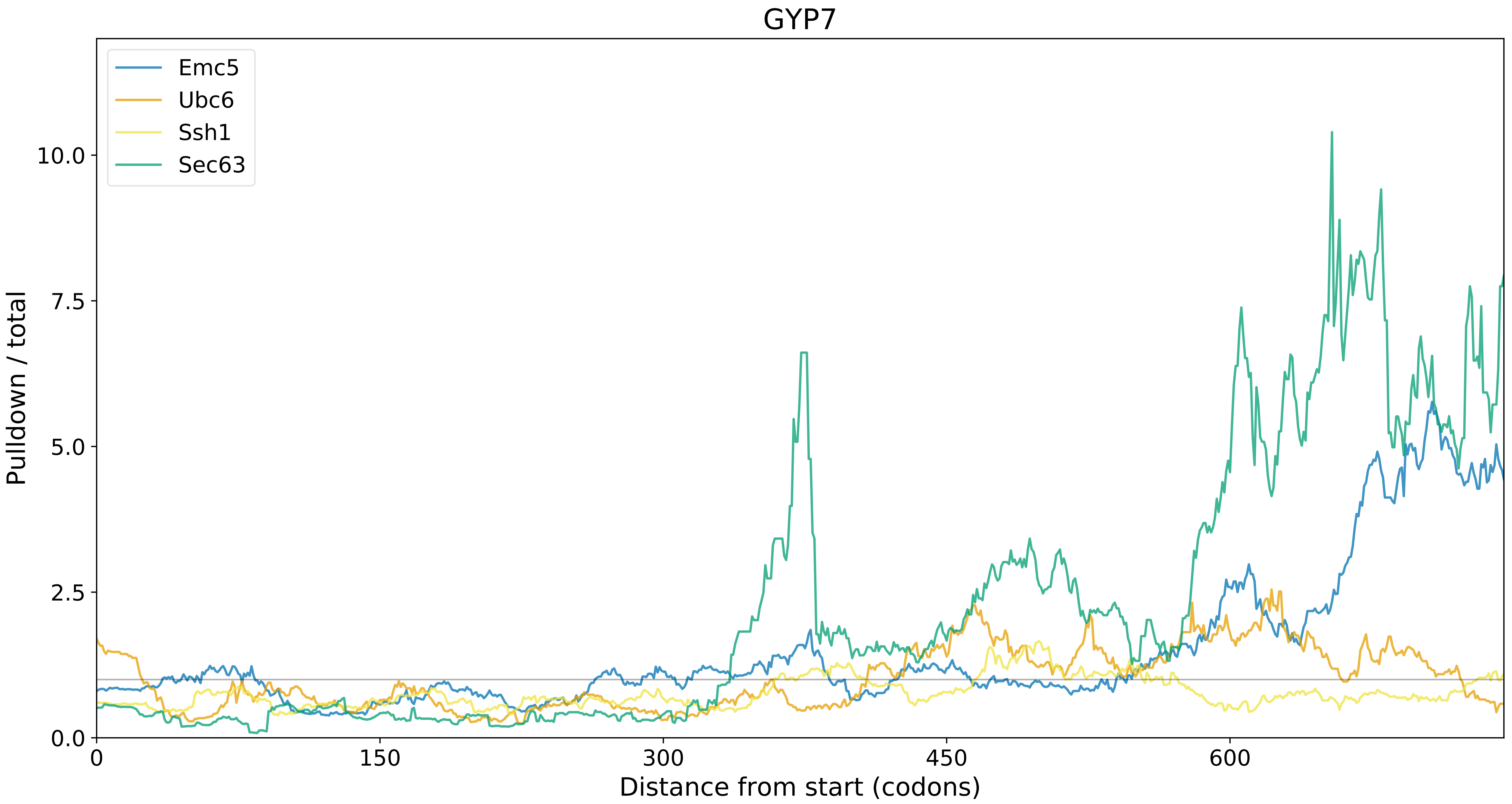
GYP7
Emc5
Ubc6
Ssh1
Sec63
Pulldown / total
0.0
2.5
5.0
7.5
10.0
0
150
300
450
600
Distance from start (codons)

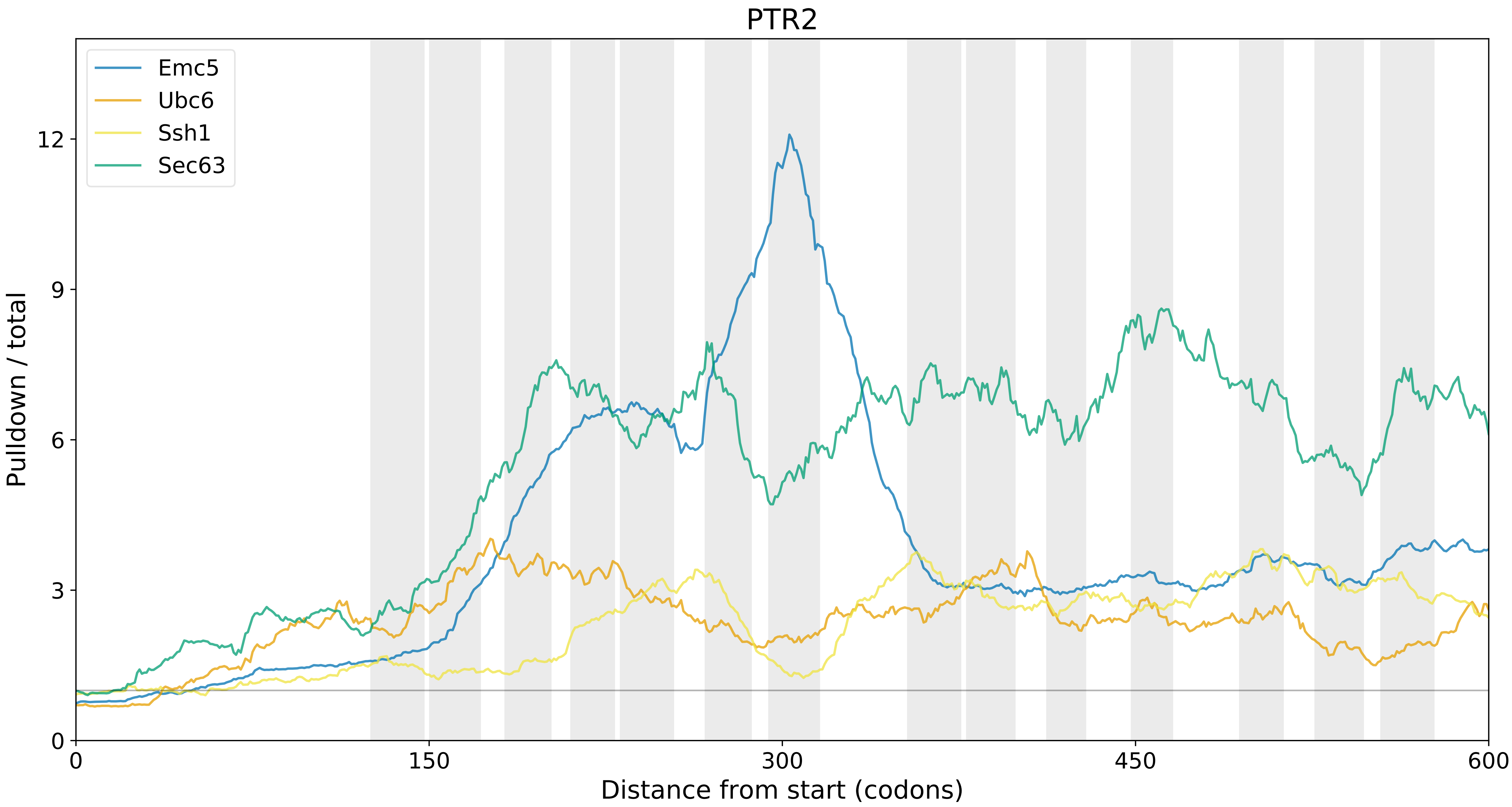

PTR2
Emc5
Ubc6
Ssh1
Sec63
Pulldown / total
Distance from start (codons)
0
3
6
9
12
0
150
300
450
600

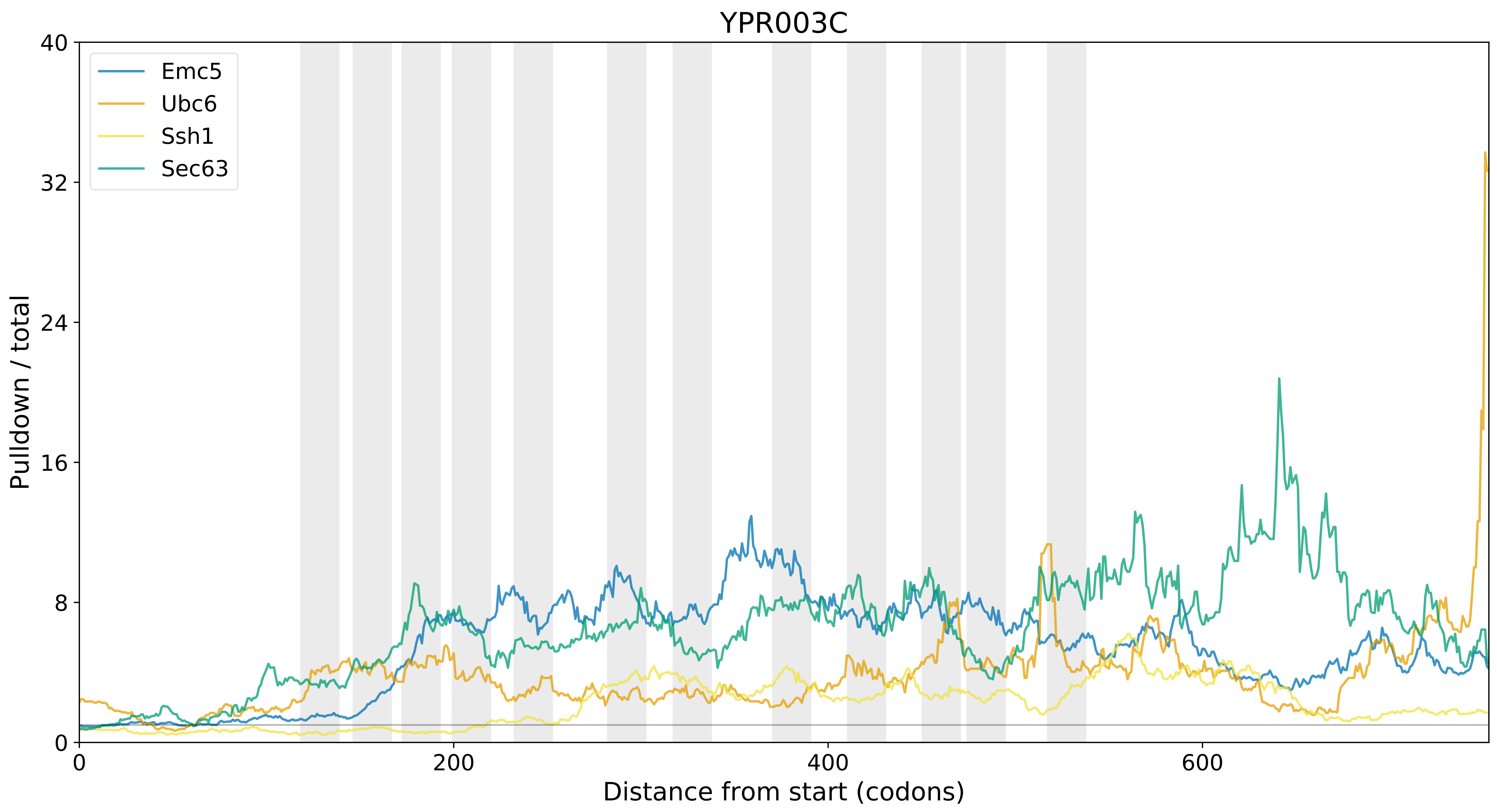

YPR003C
Emc5
Ubc6
Ssh1
Sec63
Pulldown / total
Distance from start (codons)
0
8
16
24
32
40
0
200
400
600

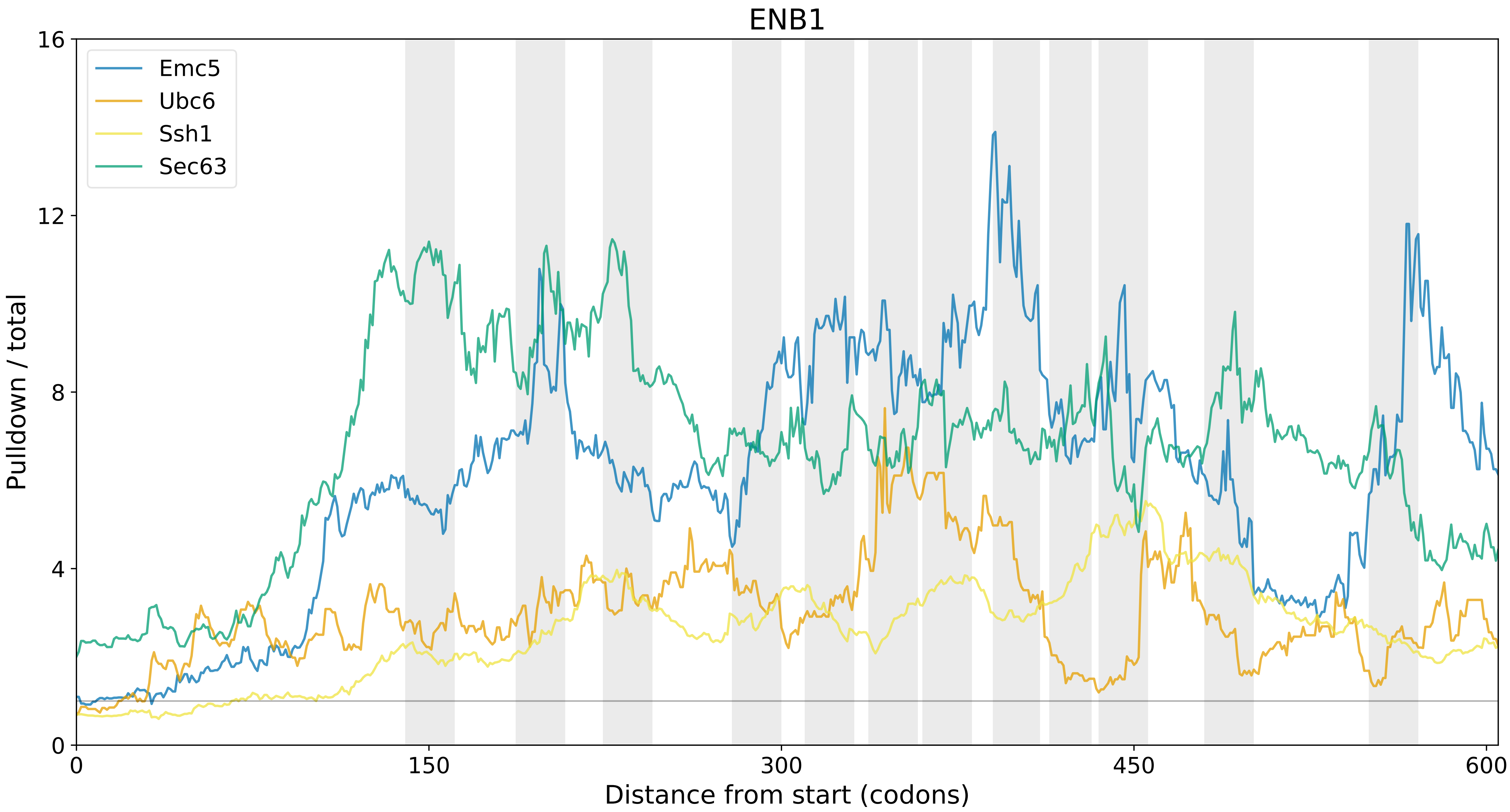
ENB1
Emc5
Ubc6
Ssh1
Sec63
Pulldown / total
16
12
8
4
0
0
150
300
450
600
Distance from start (codons)

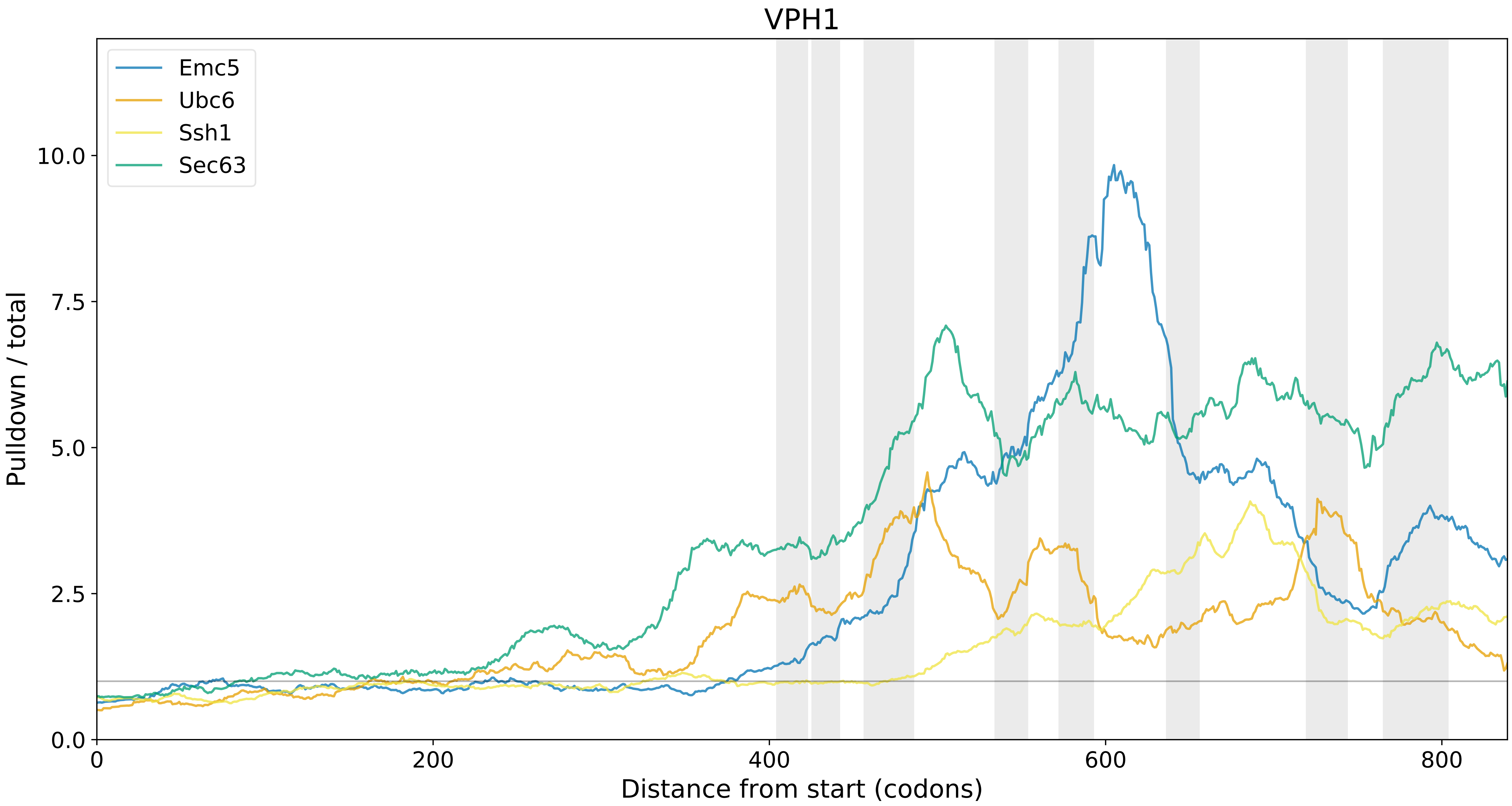

VPH1
Emc5
Ubc6
Ssh1
Sec63
Pulldown / total
10.0
7.5
5.0
2.5
0.0
0
200
400
600
800
Distance from start (codons)

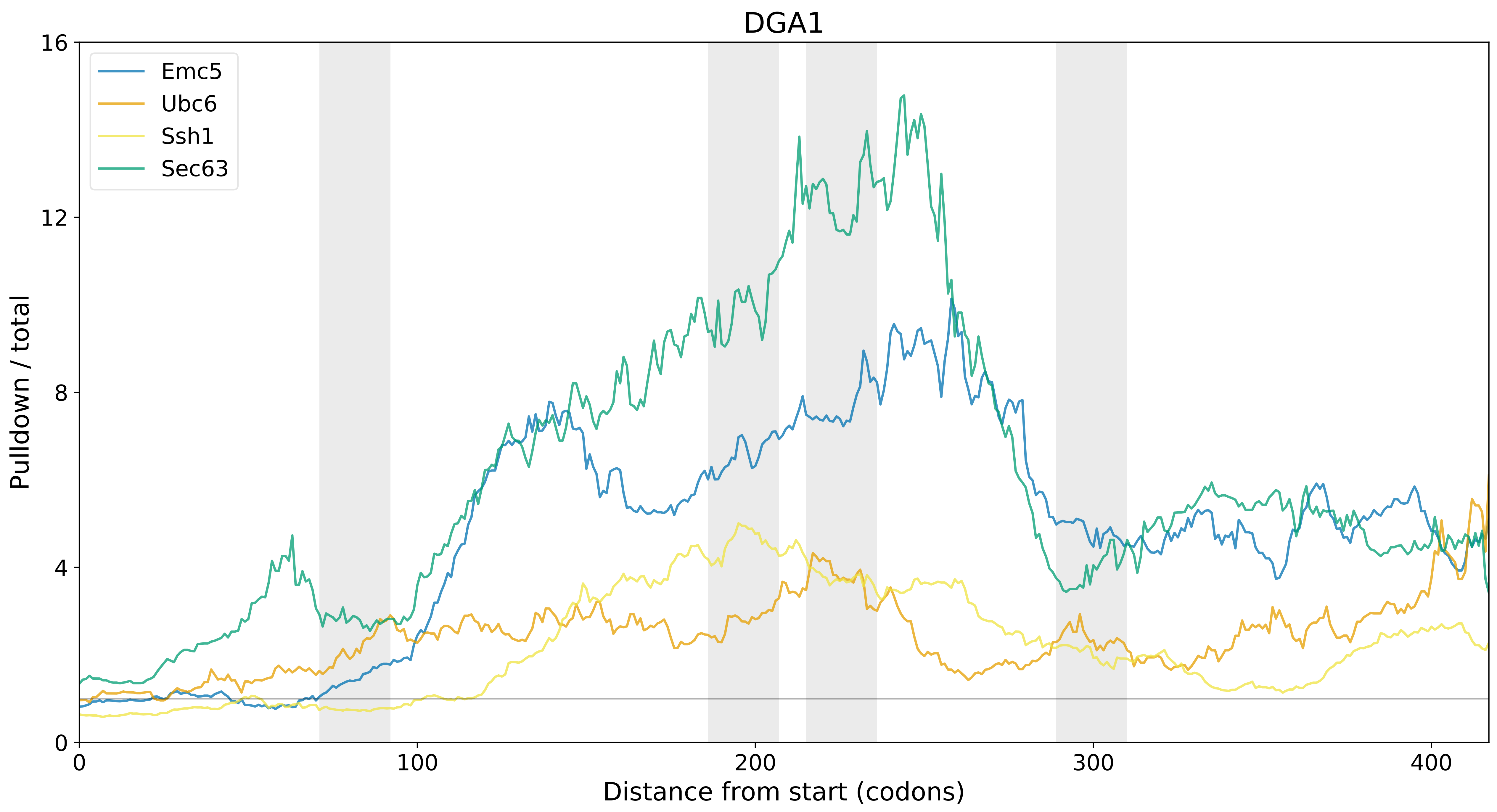

DGA1
Emc5
Ubc6
Ssh1
Sec63
Pulldown / total
Distance from start (codons)
0
4
8
12
16
0
100
200
300
400

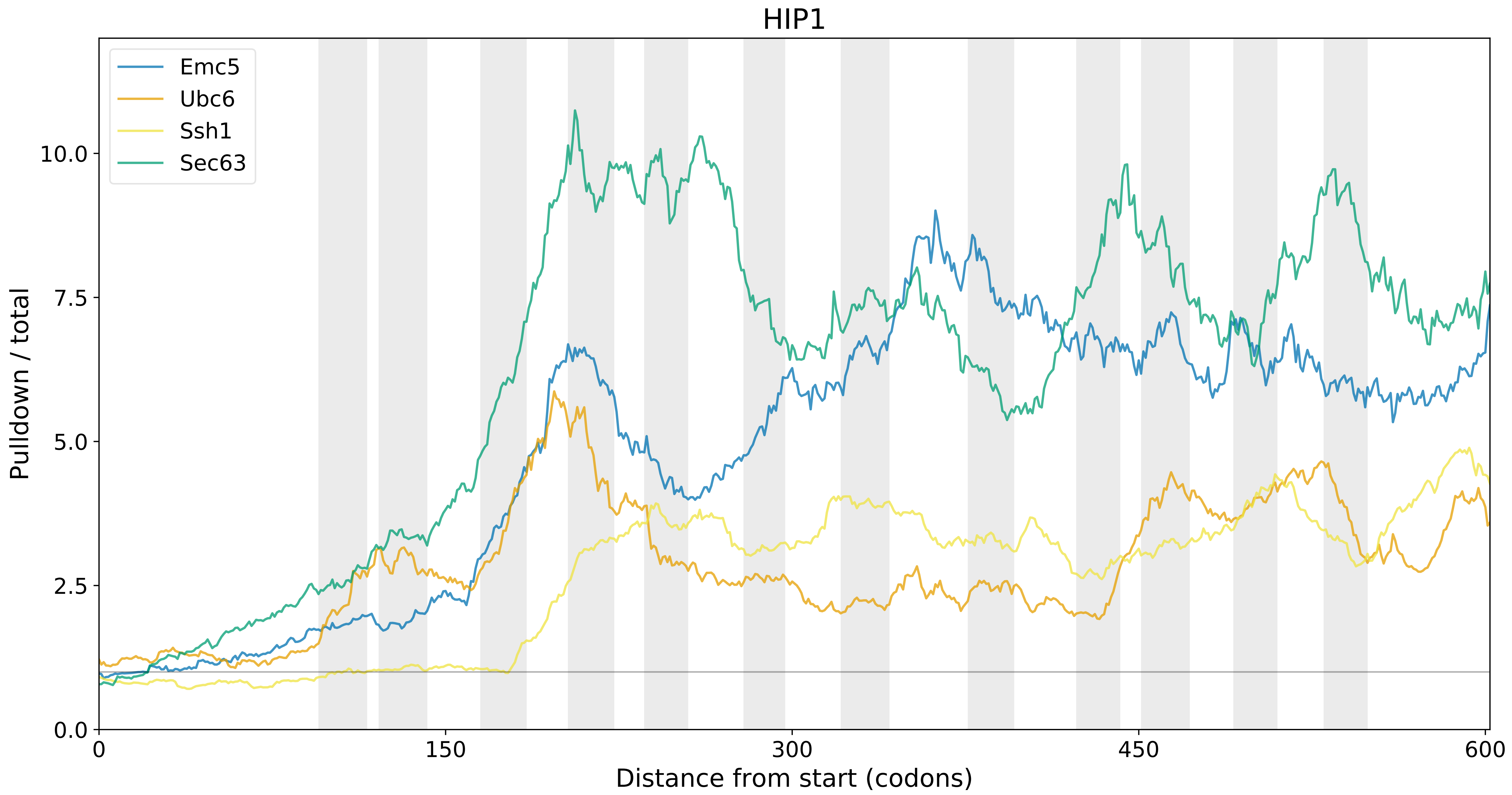
HIP1
Emc5
Ubc6
Ssh1
Sec63
Pulldown / total
10.0
7.5
5.0
2.5
0.0
0
150
300
450
600
Distance from start (codons)

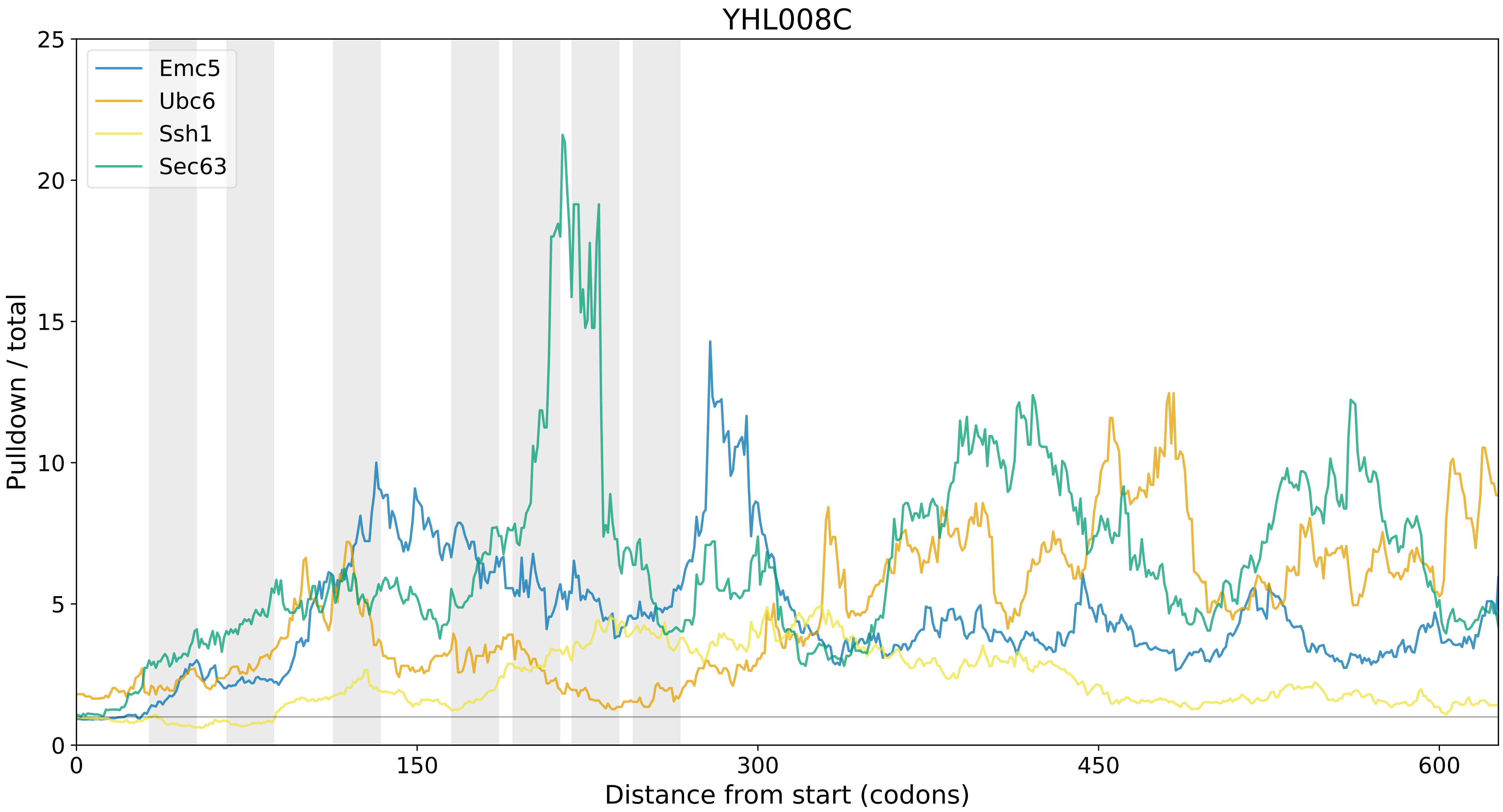
YHL008C
Emc5
Ubc6
Ssh1
Sec63
Pulldown / total
25
20
15
10
5
0
0
150
300
450
600
Distance from start (codons)

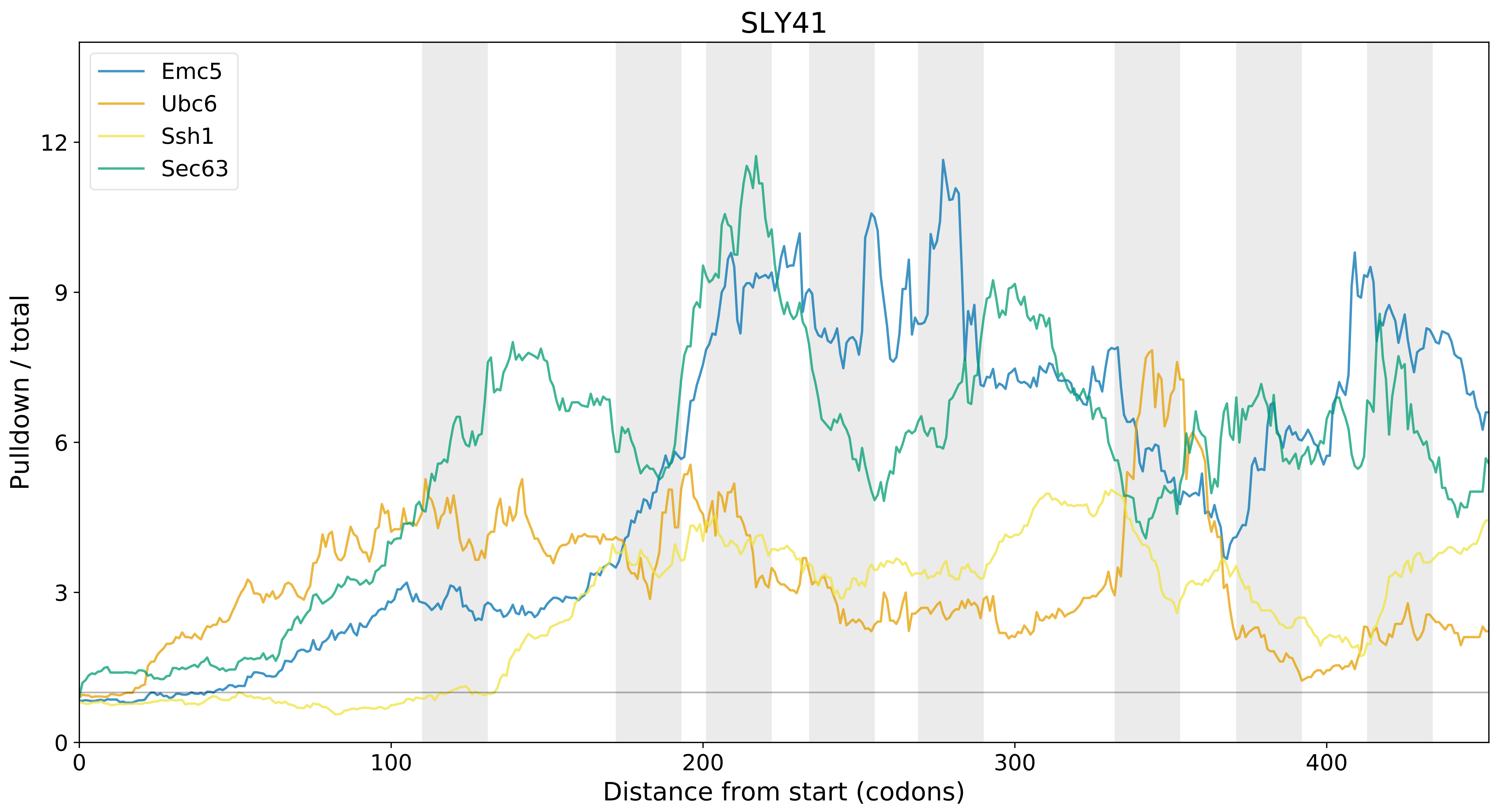
SLY41
Emc5
Ubc6
Ssh1
Sec63
Pulldown / total
0
3
6
9
12
0
100
200
300
400
Distance from start (codons)

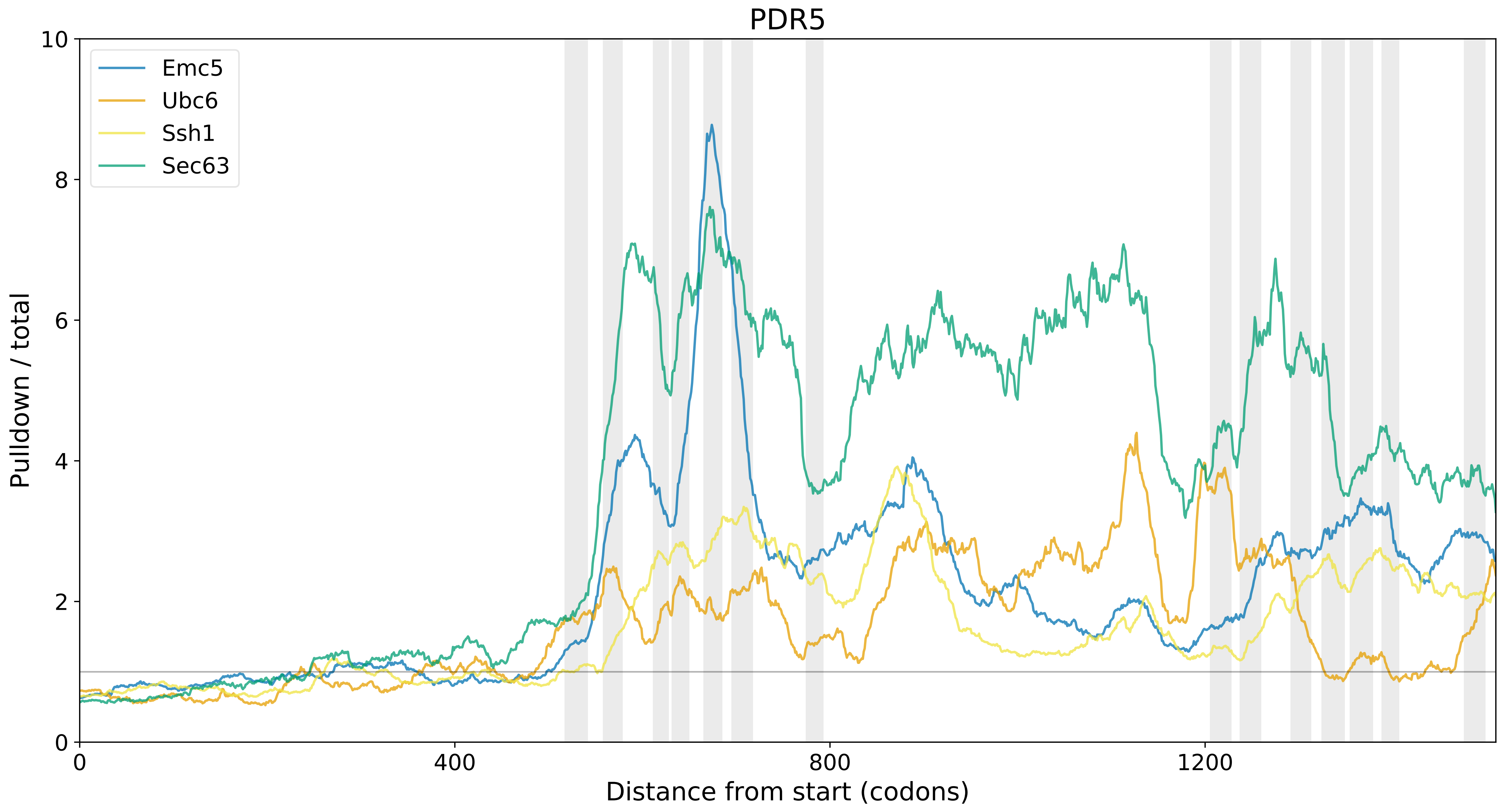

PDR5
Emc5
Ubc6
Ssh1
Sec63
Pulldown / total
0
2
4
6
8
10
0
400
800
1200
Distance from start (codons)

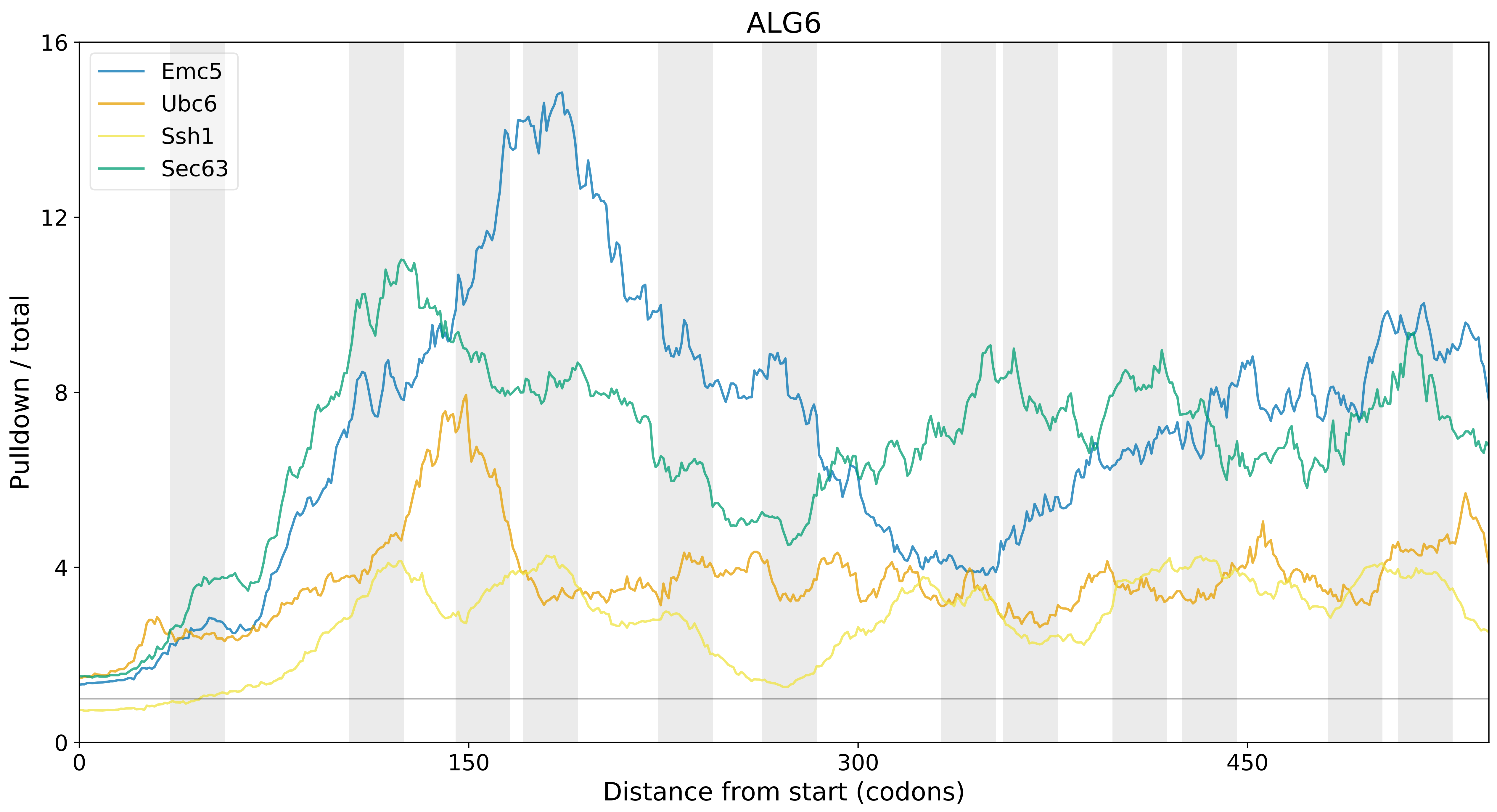

ALG6
Emc5
Ubc6
Ssh1
Sec63
Pulldown / total
Distance from start (codons)
0
4
8
12
16
0
150
300
450

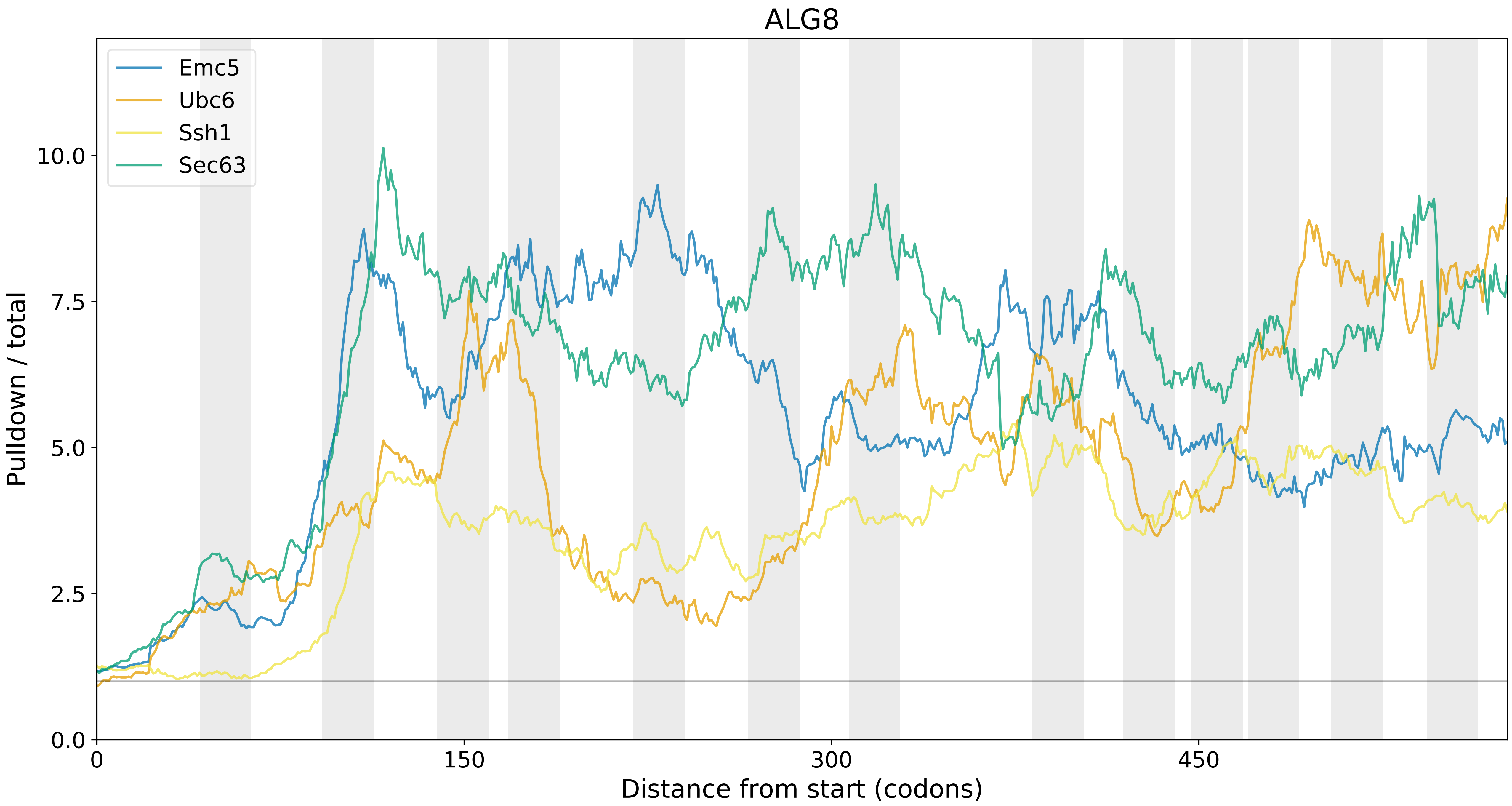

ALG8
Emc5
Ubc6
Ssh1
Sec63
Pulldown / total
0.0
2.5
5.0
7.5
10.0
0
150
300
450
Distance from start (codons)

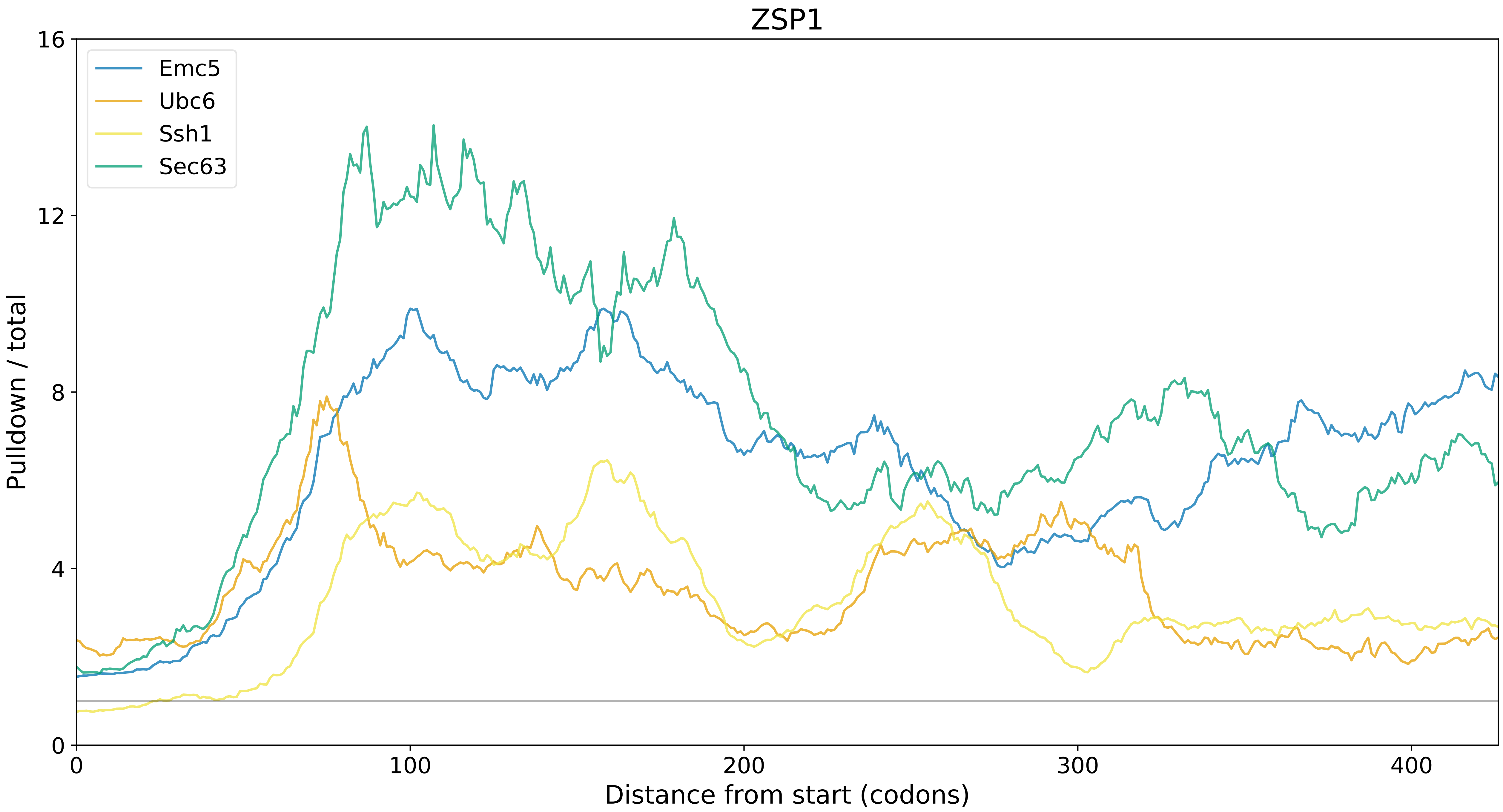
ZSP1
Emc5
Ubc6
Ssh1
Sec63
Pulldown / total
Distance from start (codons)
0
4
8
12
16
0
100
200
300
400

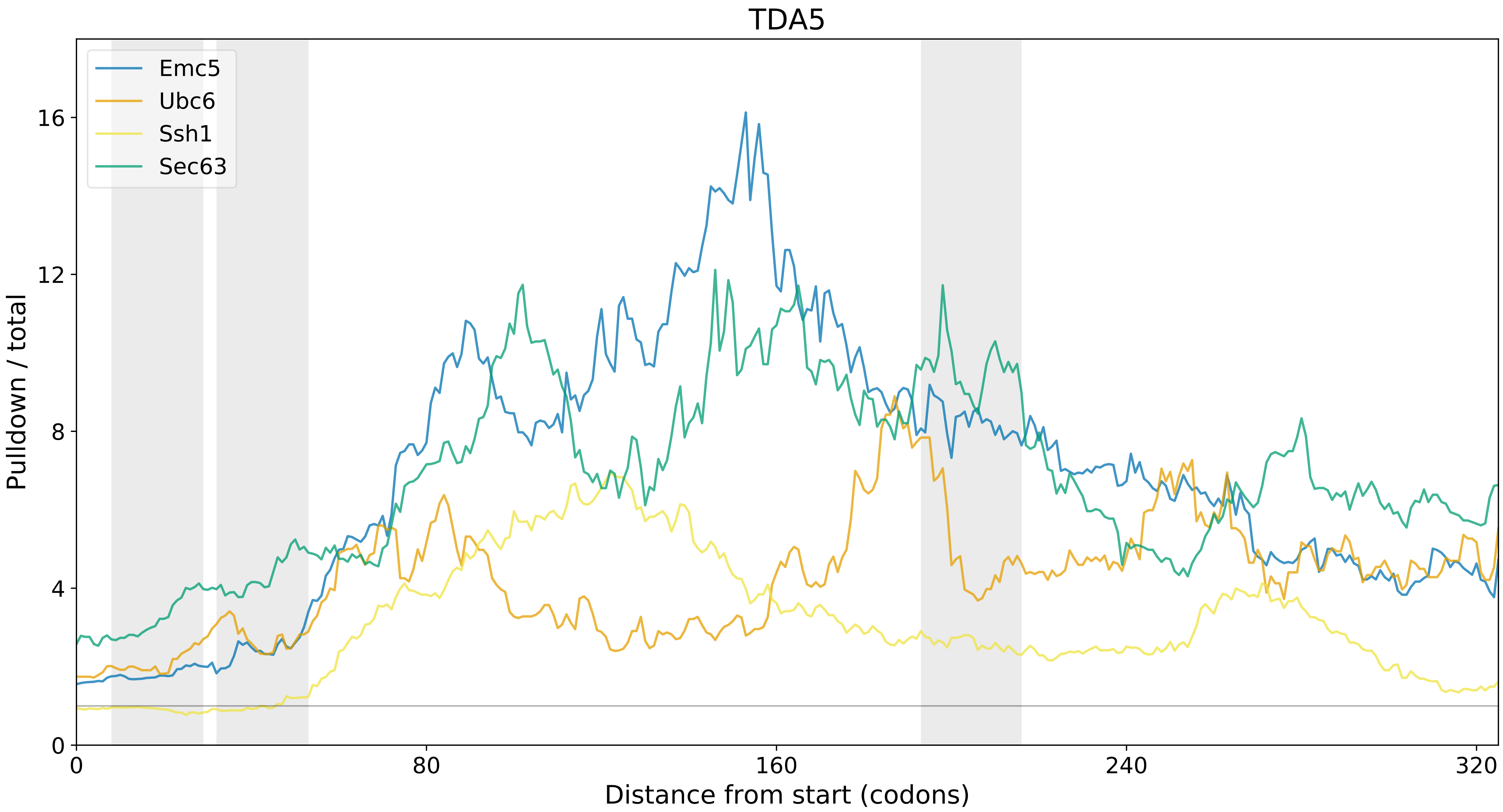

TDA5
Emc5
Ubc6
Ssh1
Sec63
Pulldown / total
0
4
8
12
16
0
80
160
240
320
Distance from start (codons)

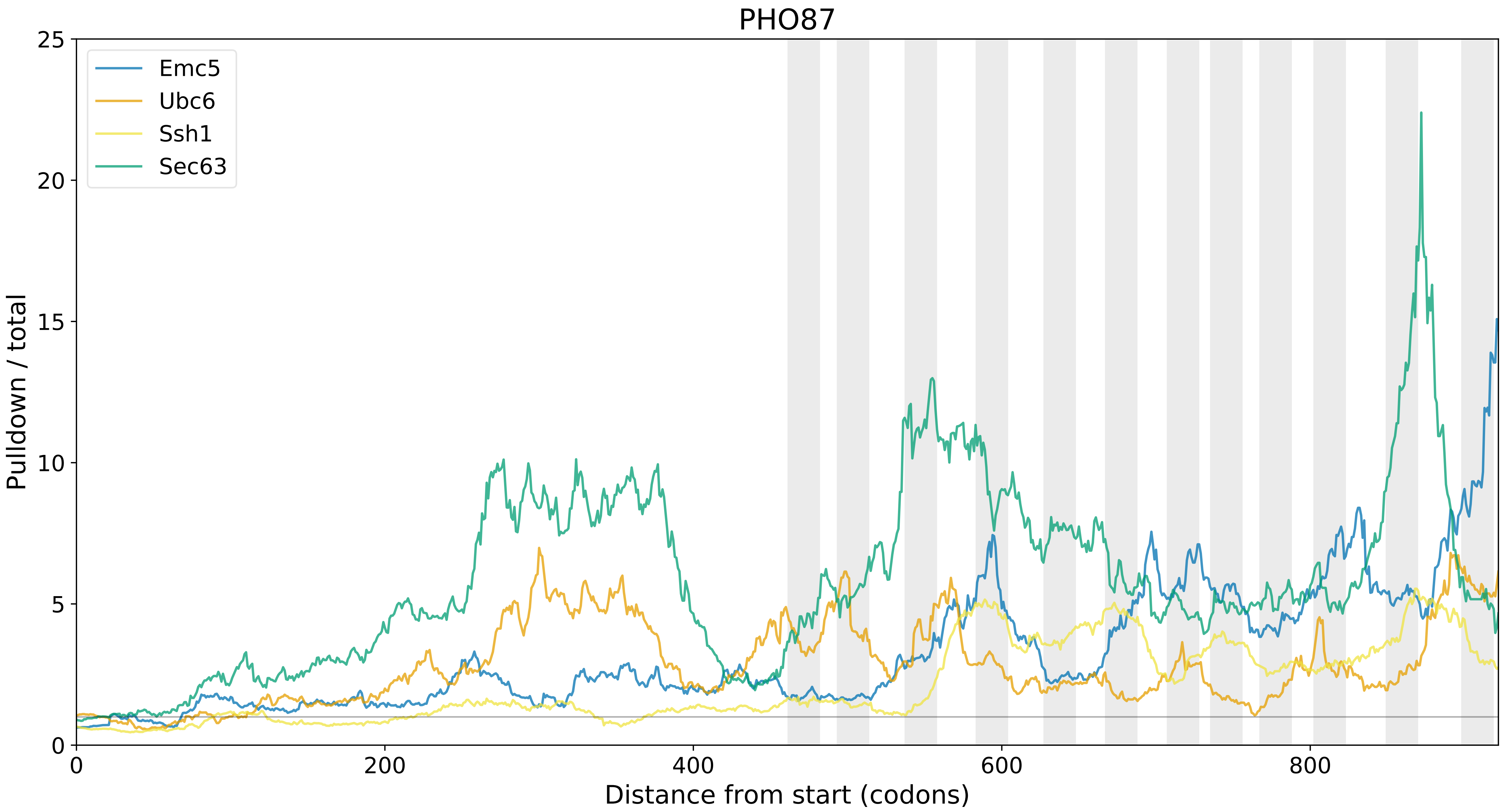

PHO87
Emc5
Ubc6
Ssh1
Sec63
Pulldown / total
0
5
10
15
20
25
0
200
400
600
800
Distance from start (codons)

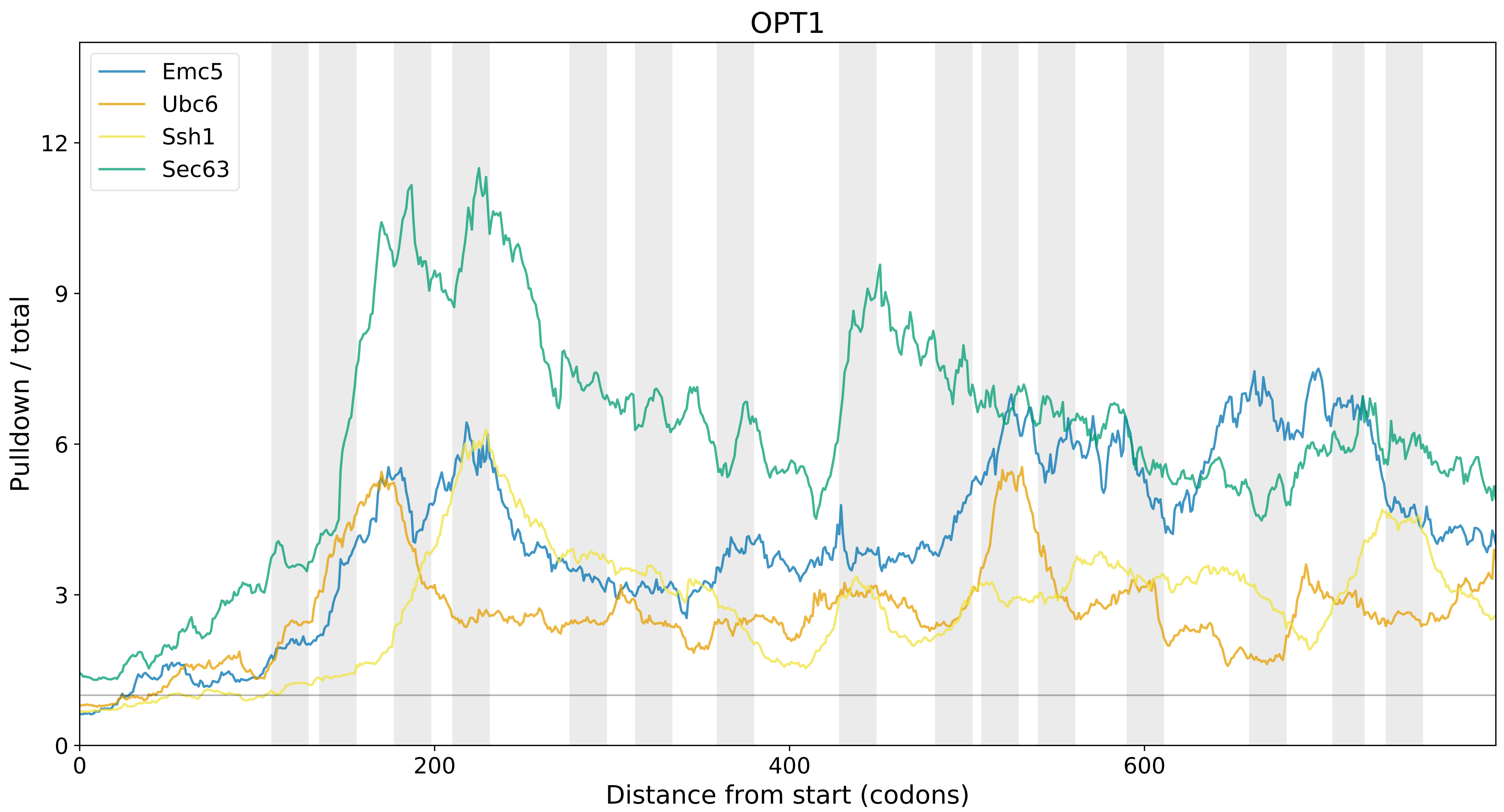
OPT1
Emc5
Ubc6
Ssh1
Sec63
Pulldown / total
Distance from start (codons)
0
3
6
9
12
0
200
400
600

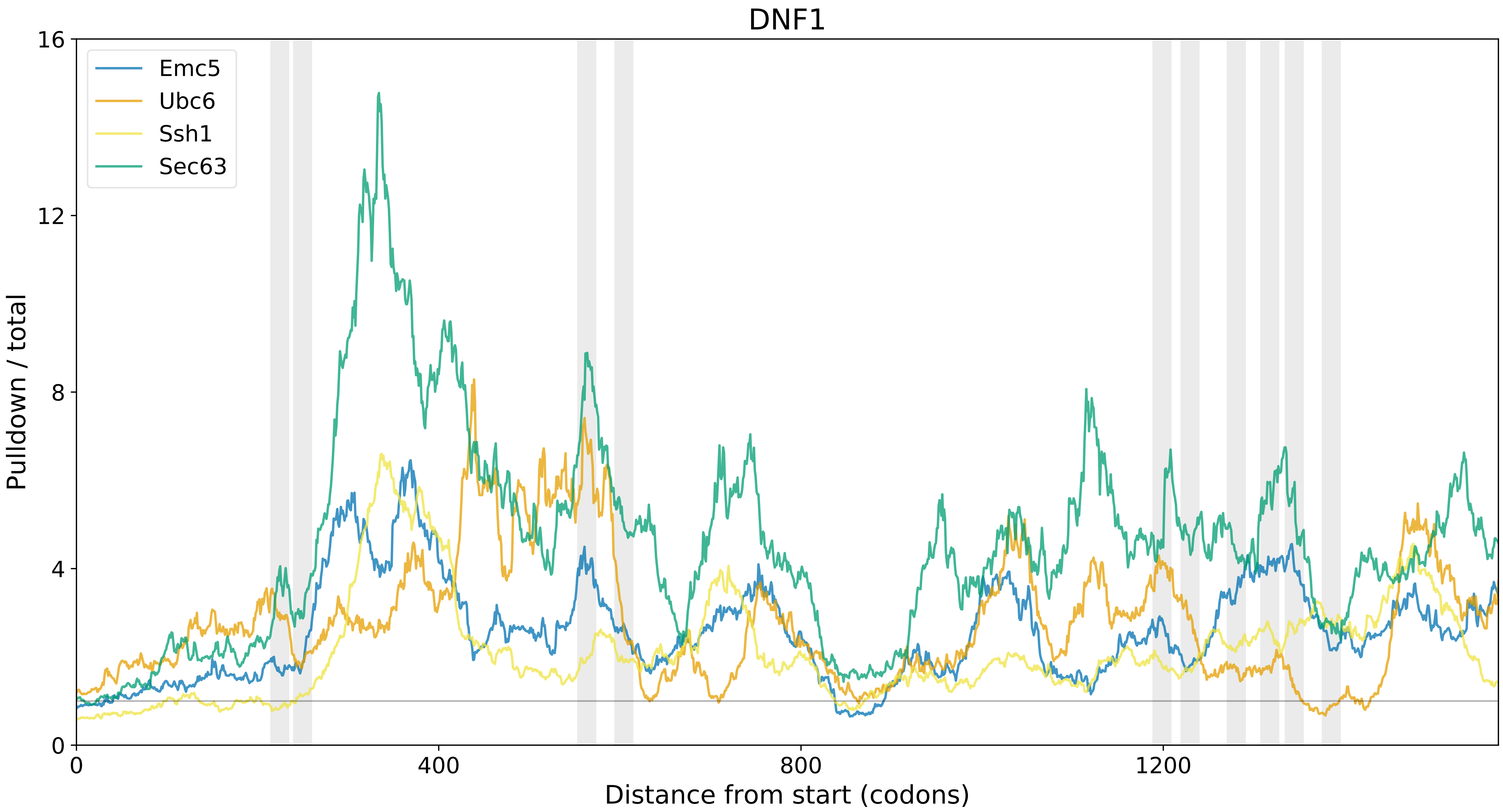
DNF1
Emc5
Ubc6
Ssh1
Sec63
Pulldown / total
Distance from start (codons)
0
4
8
12
16
0
400
800
1200

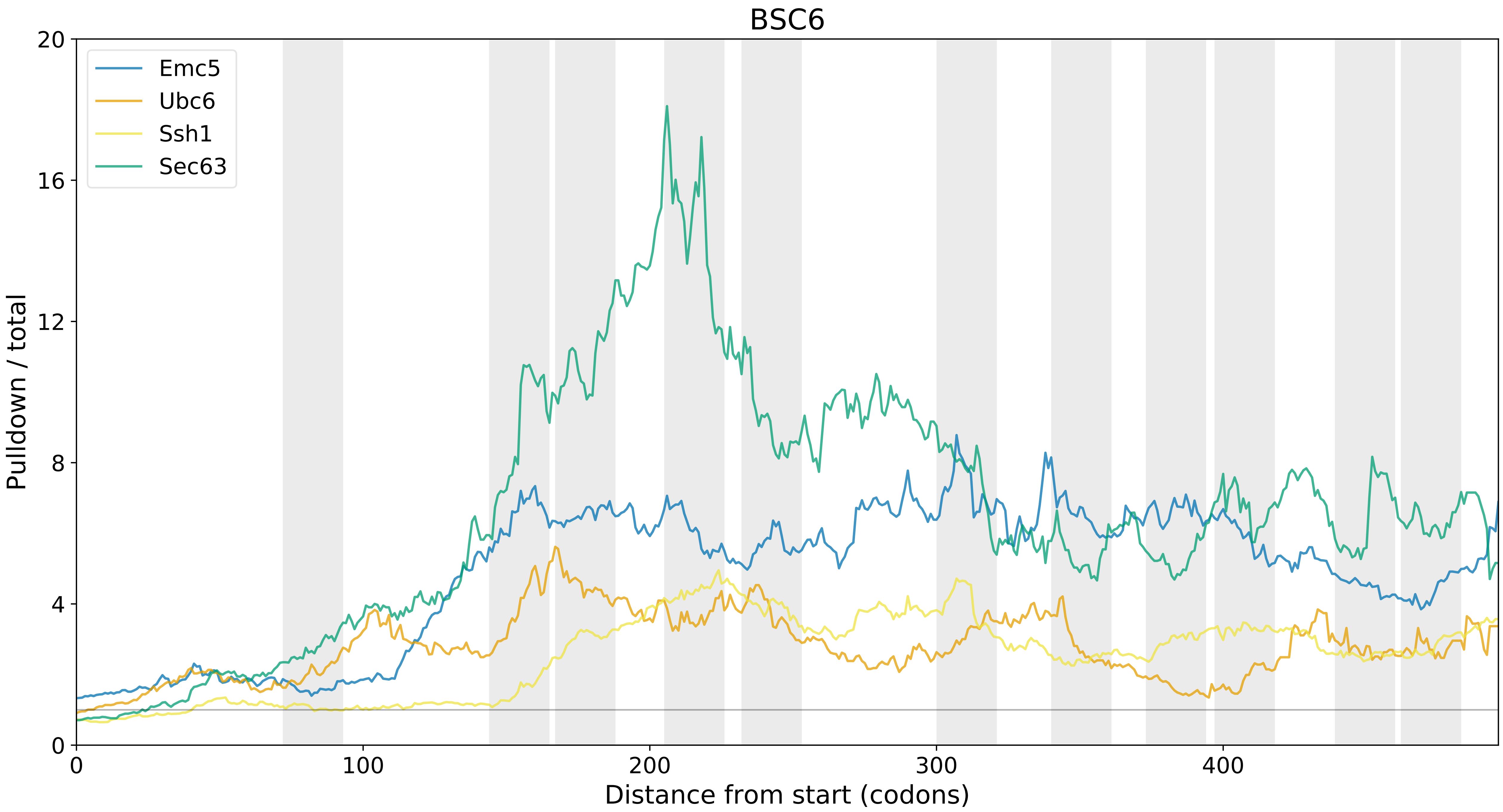
BSC6
Emc5
Ubc6
Ssh1
Sec63
Pulldown / total
Distance from start (codons)
0
4
8
12
16
20
0
100
200
300
400

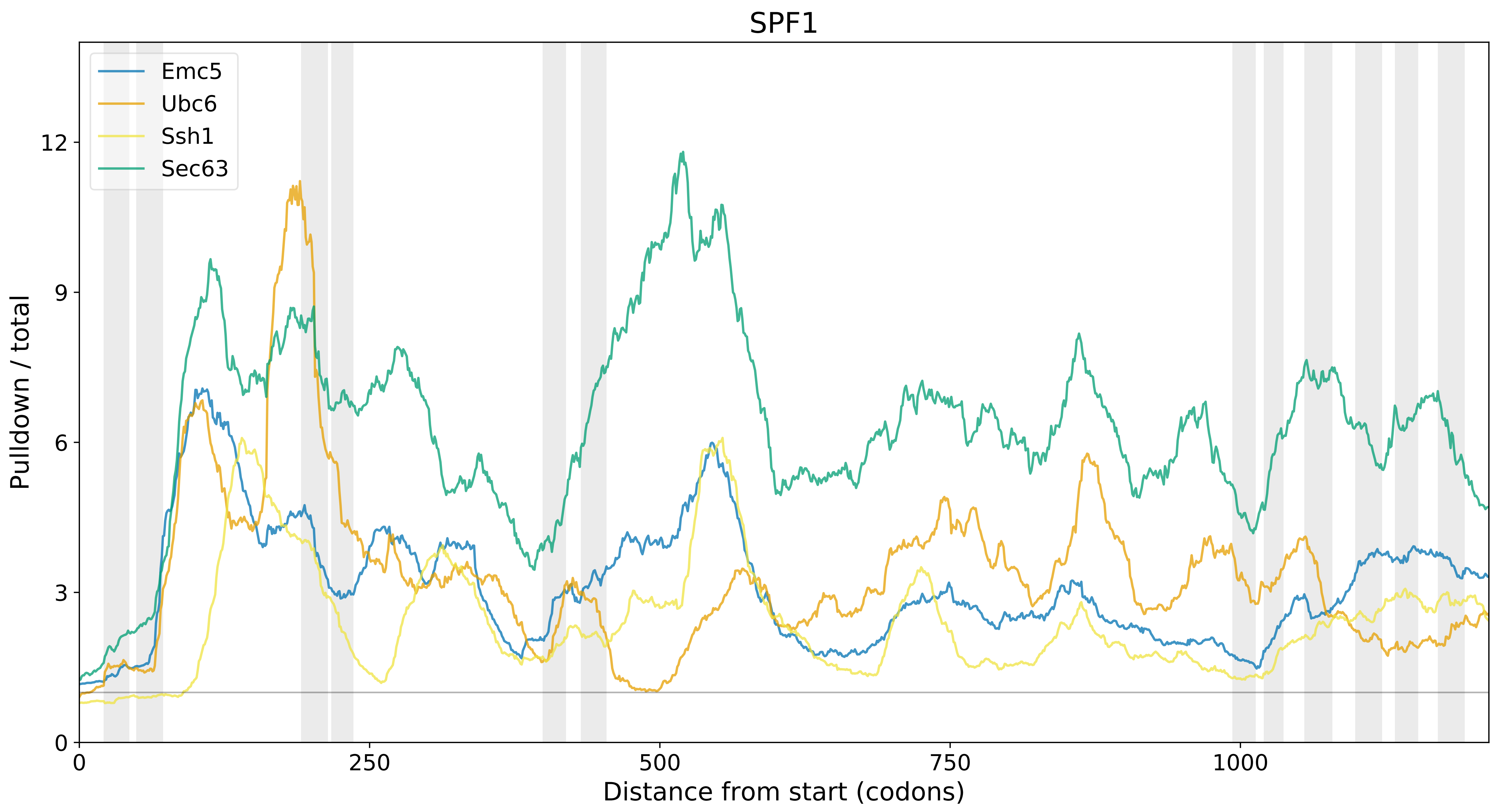

SPF1
Emc5
Ubc6
Ssh1
Sec63
Pulldown / total
0
3
6
9
12
0
250
500
750
1000
Distance from start (codons)

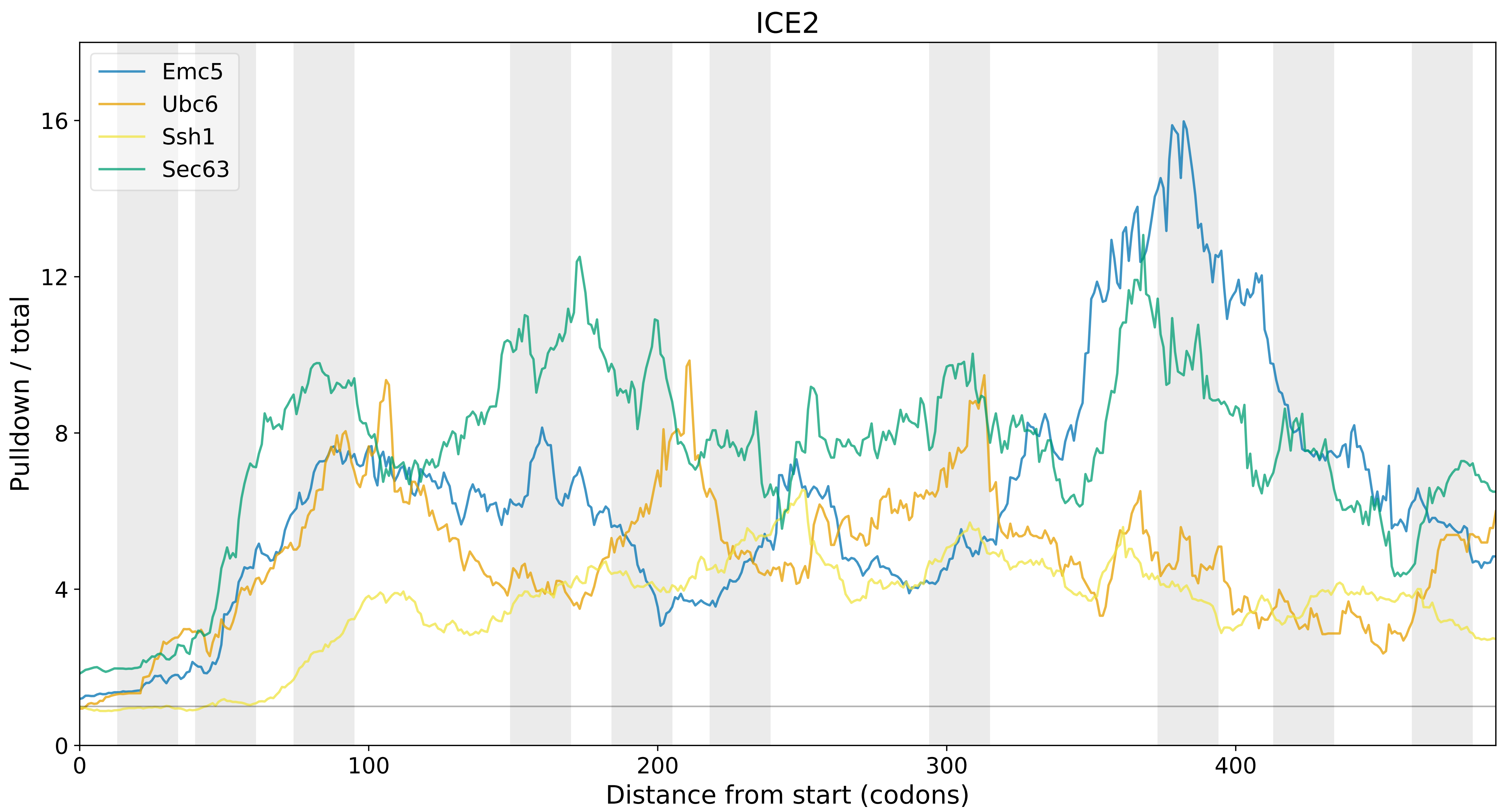
ICE2
Emc5
Ubc6
Ssh1
Sec63
Pulldown / total
16
12
8
4
0
0
100
200
300
400
Distance from start (codons)

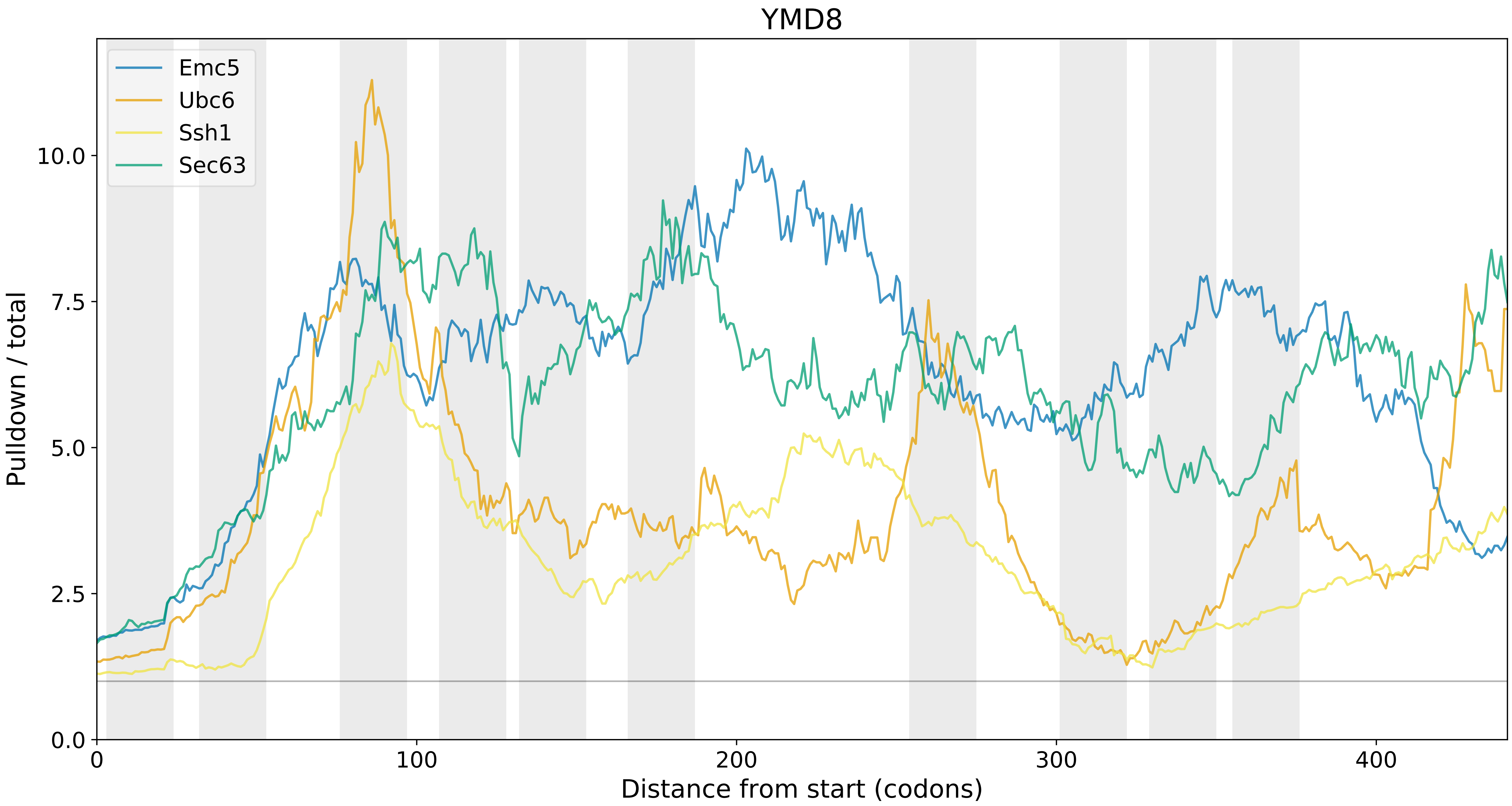
YMD8
Emc5
Ubc6
Ssh1
Sec63
Pulldown / total
10.0
7.5
5.0
2.5
0.0
0
100
200
300
400
Distance from start (codons)

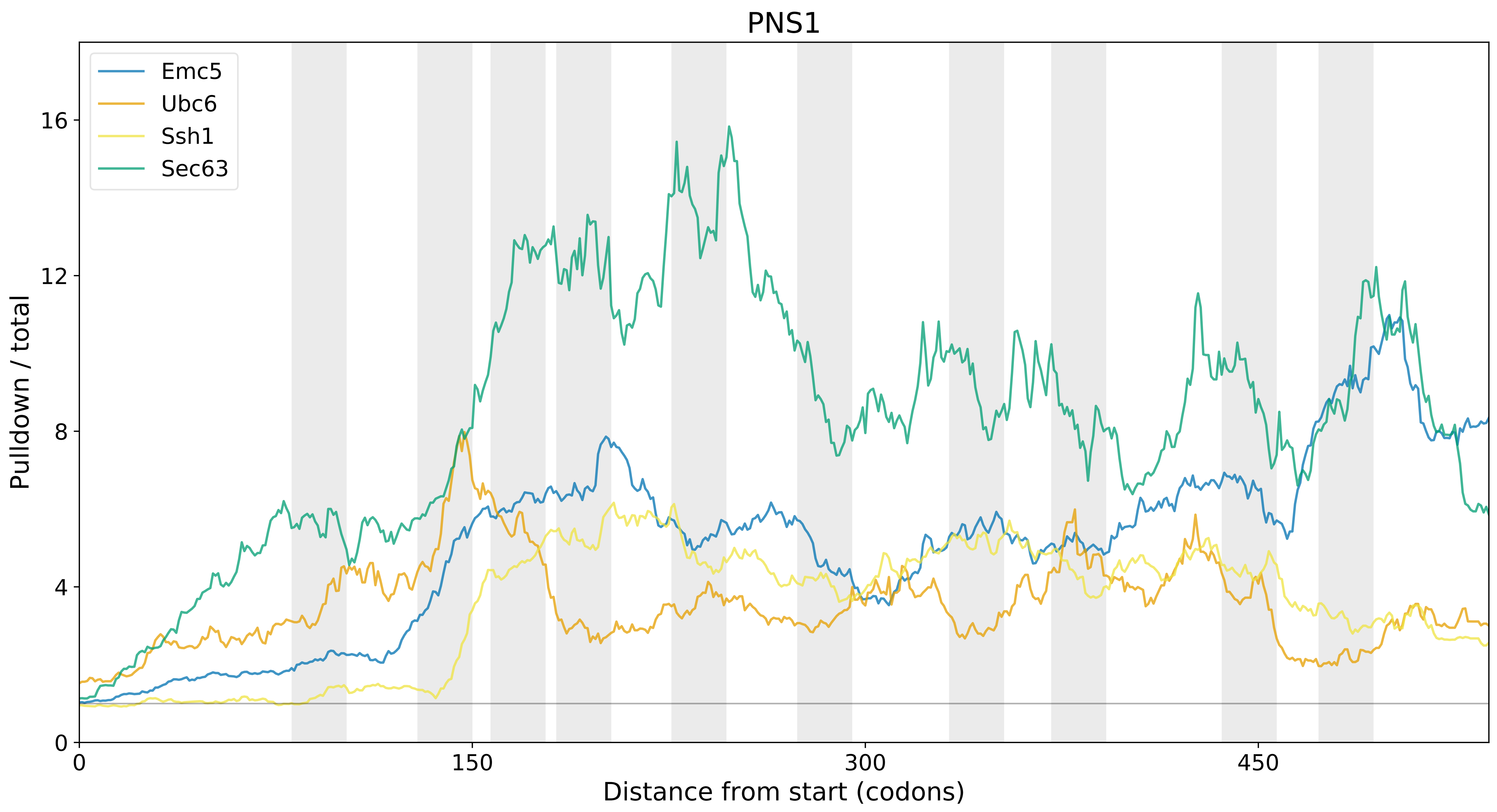
PNS1
Emc5
Ubc6
Ssh1
Sec63
Pulldown / total
0
4
8
12
16
0
150
300
450
Distance from start (codons)

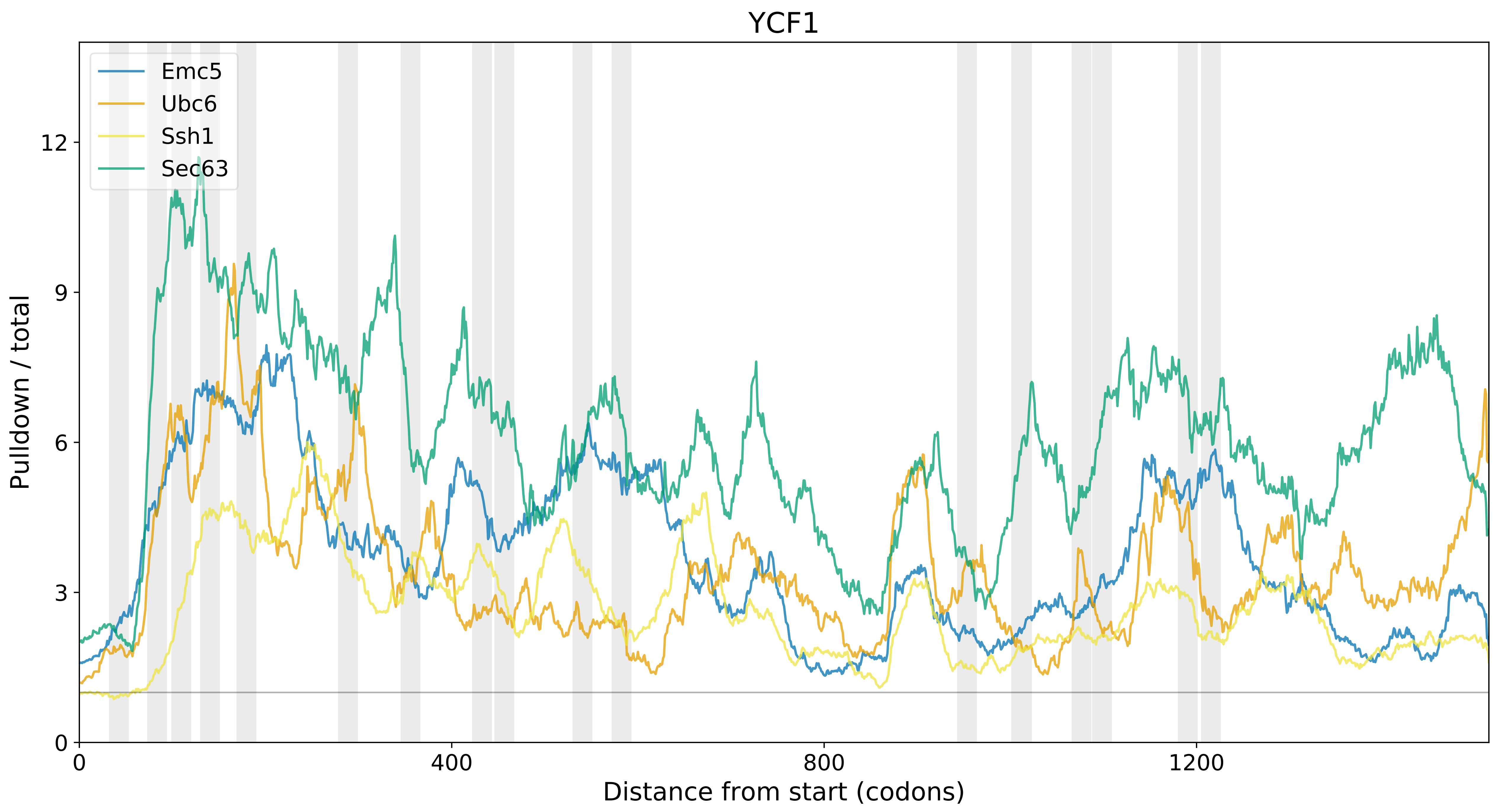
YCF1
Emc5
Ubc6
Ssh1
Sec63
Pulldown / total
0
3
6
9
12
0
400
800
1200
Distance from start (codons)

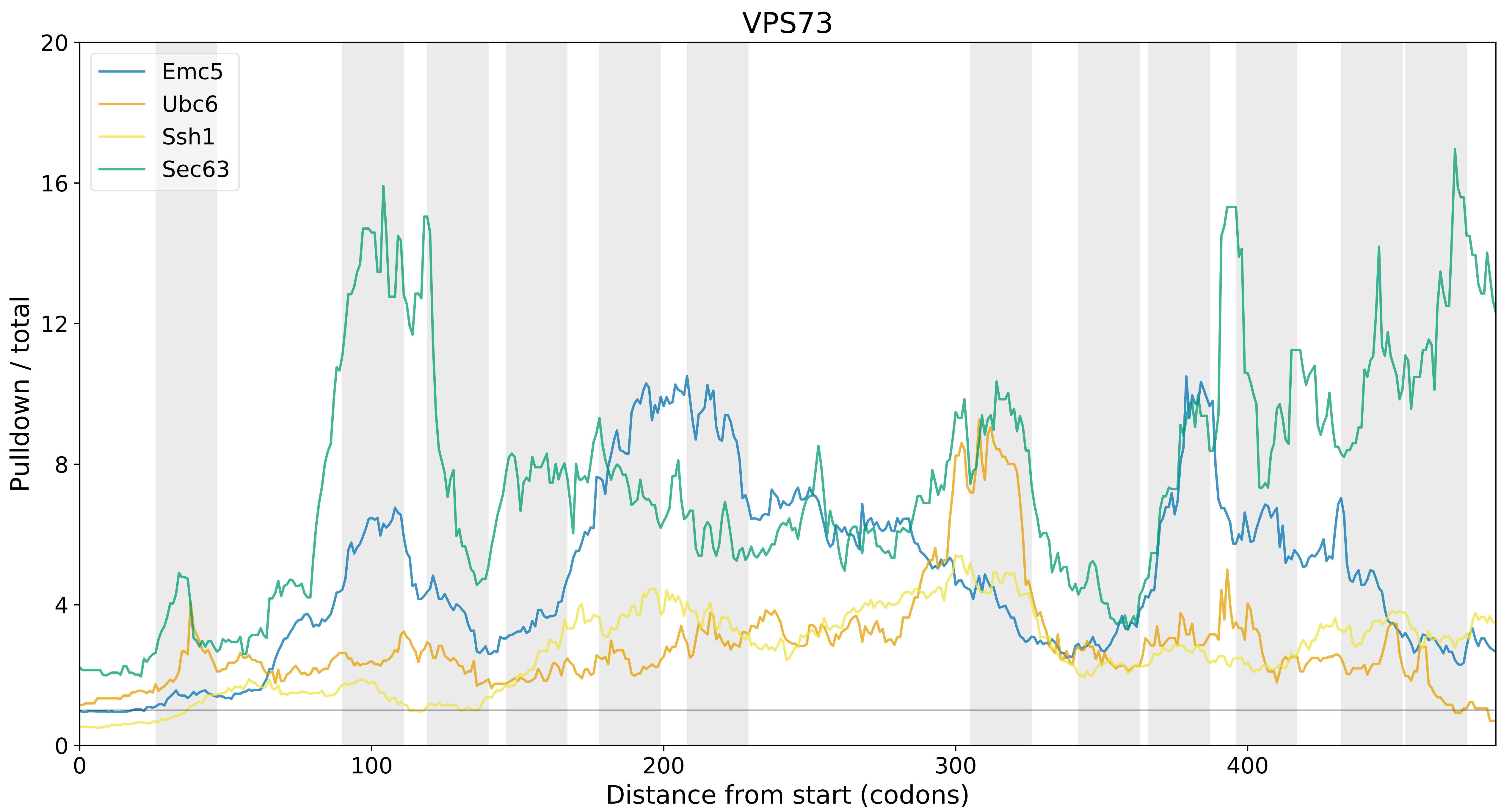

VPS73
Emc5
Ubc6
Ssh1
Sec63
Pulldown / total
Distance from start (codons)
0
4
8
12
16
20
0
100
200
300
400

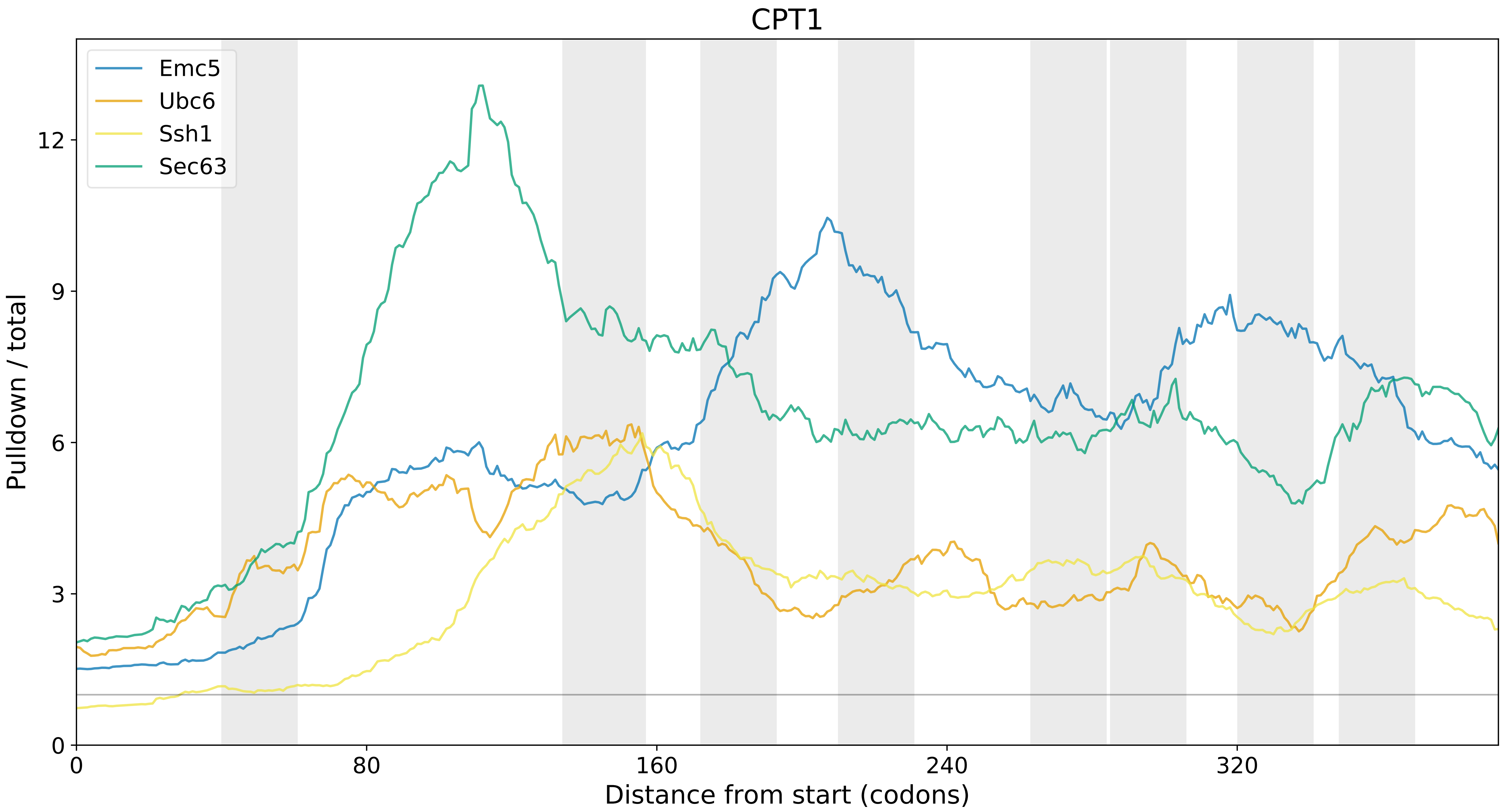
CPT1
Emc5
Ubc6
Ssh1
Sec63
Pulldown / total
0
3
6
9
12
0
80
160
240
320
Distance from start (codons)

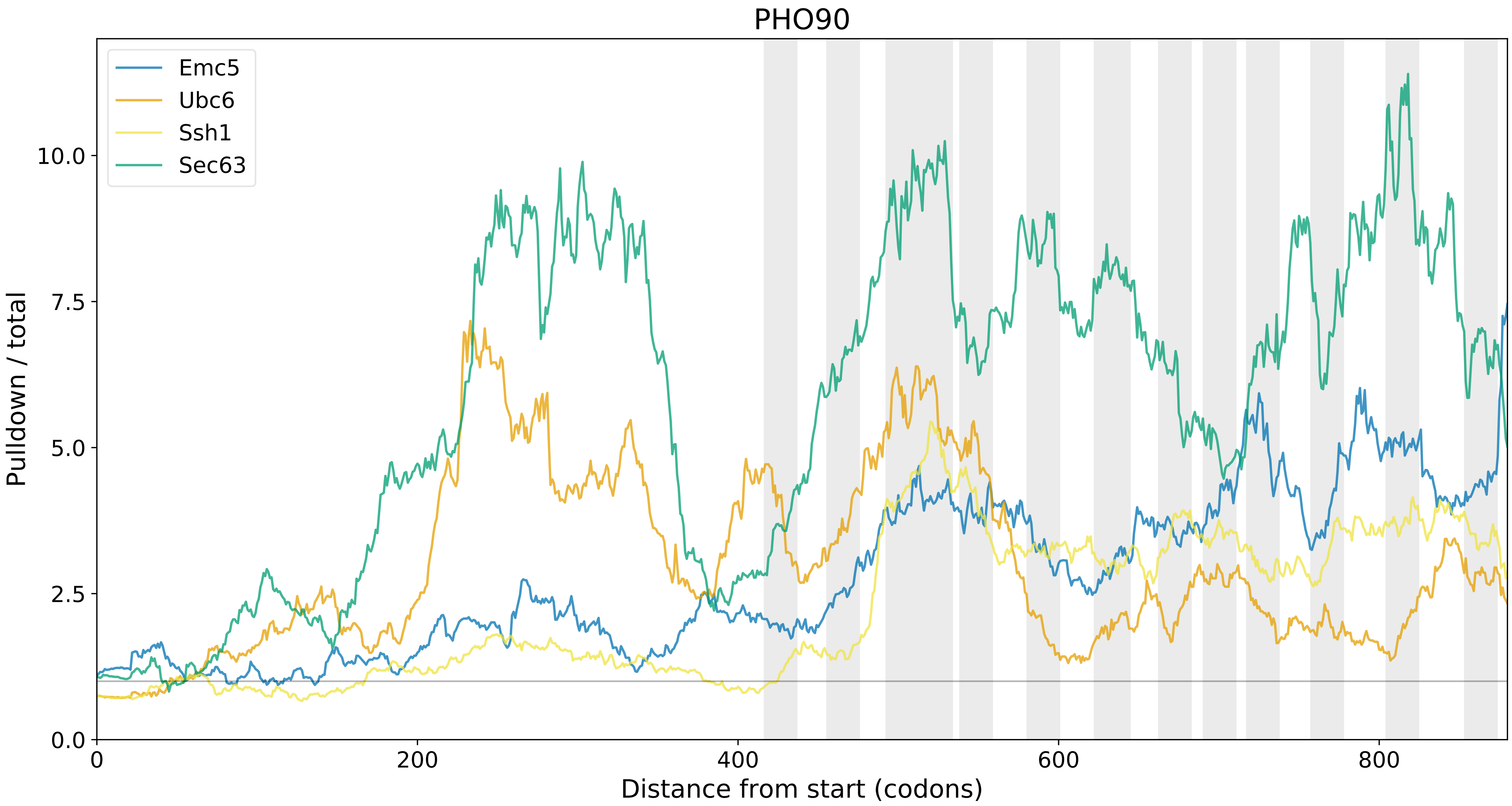
PHO90
Emc5
Ubc6
Ssh1
Sec63
Pulldown / total
Distance from start (codons)
0.0
2.5
5.0
7.5
10.0
0
200
400
600
800

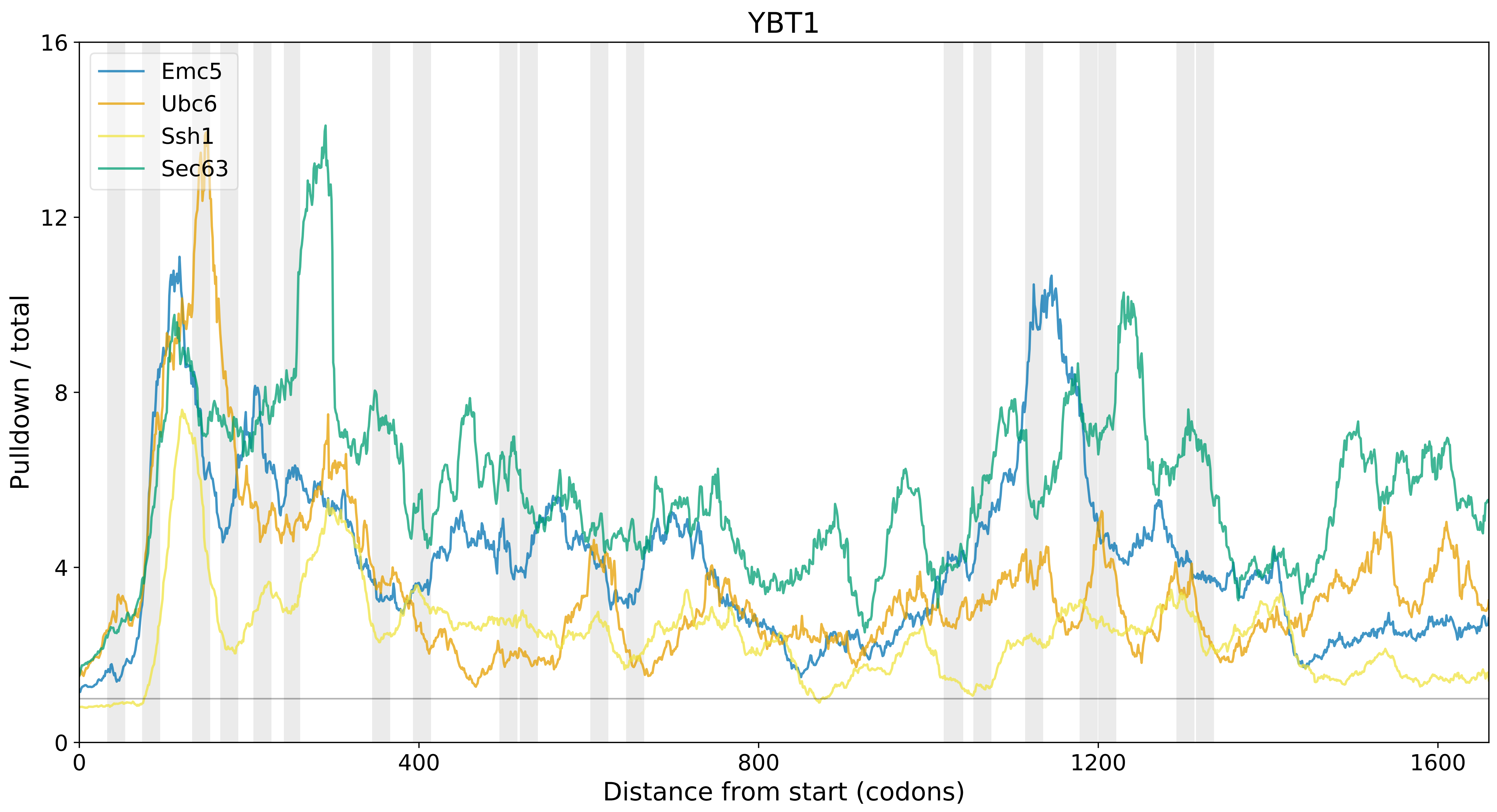

YBT1
Emc5
Ubc6
Ssh1
Sec63
Pulldown / total
Distance from start (codons)
0
4
8
12
16
0
400
800
1200
1600

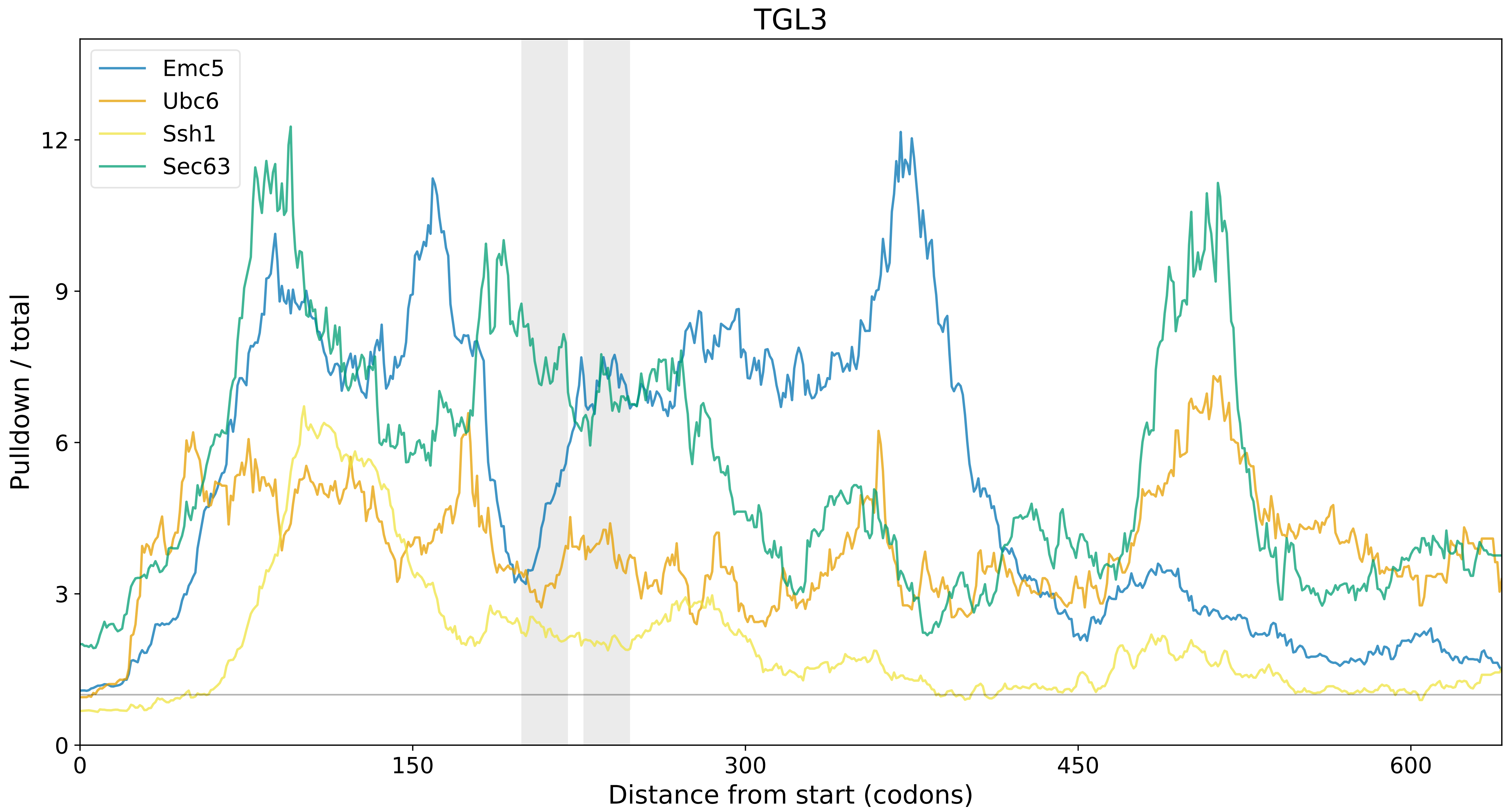
TGL3
Emc5
Ubc6
Ssh1
Sec63
Pulldown / total
Distance from start (codons)
0
3
6
9
12
0
150
300
450
600

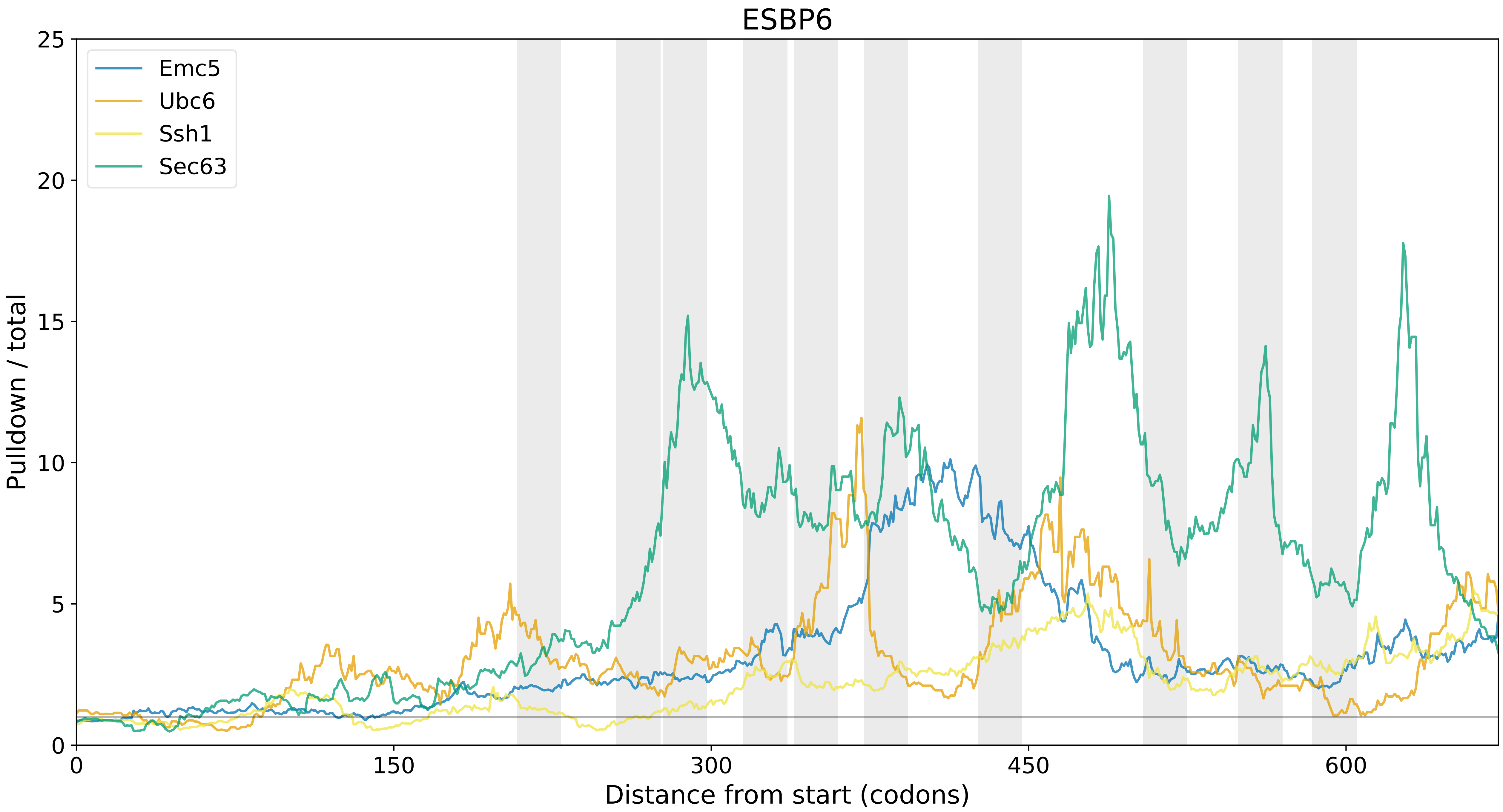

ESBP6
Emc5
Ubc6
Ssh1
Sec63
Pulldown / total
Distance from start (codons)
0
5
10
15
20
25
0
150
300
450
600

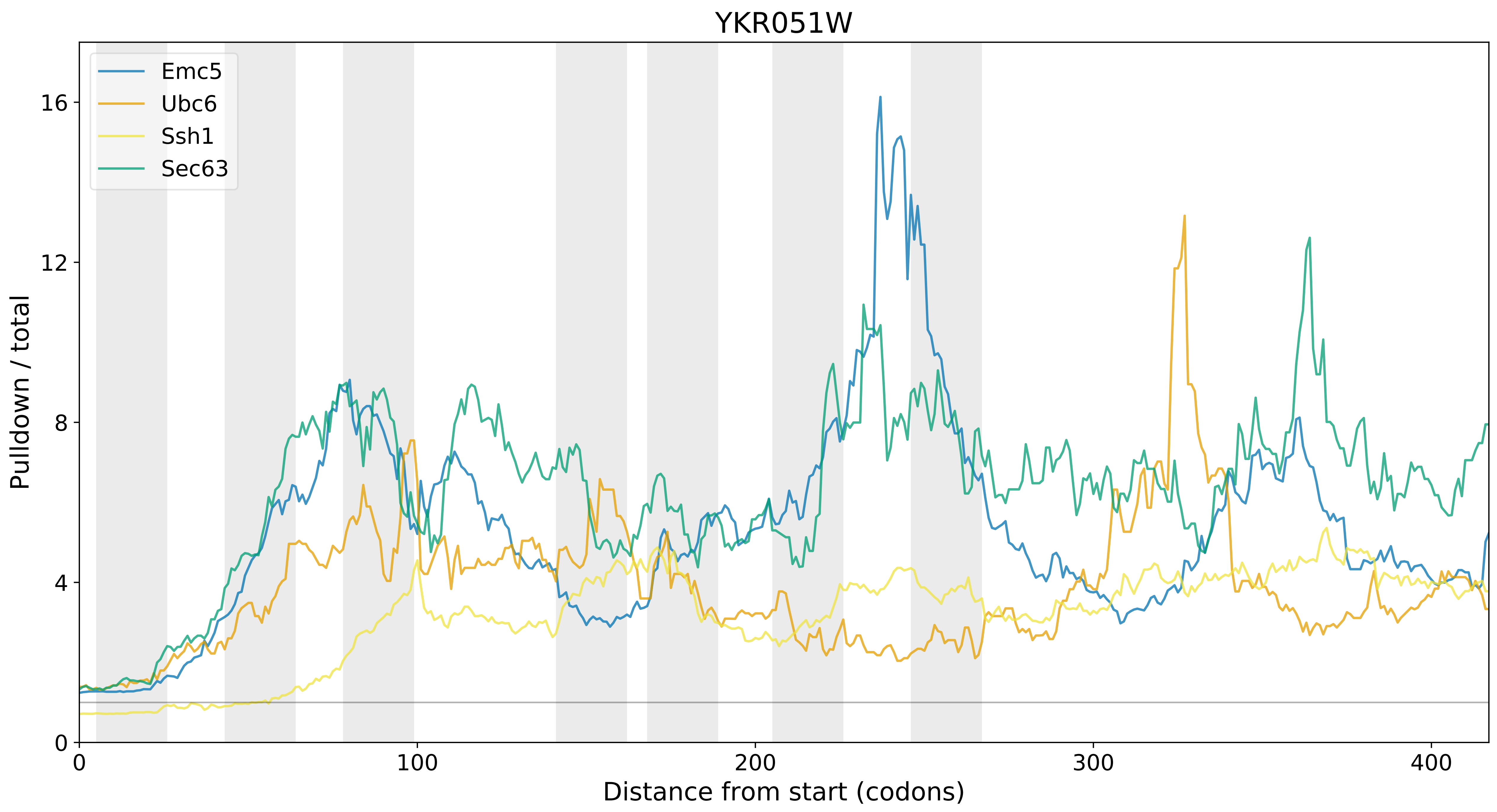

YKR051W
Emc5
Ubc6
Ssh1
Sec63
Pulldown / total
0
4
8
12
16
0
100
200
300
400
Distance from start (codons)

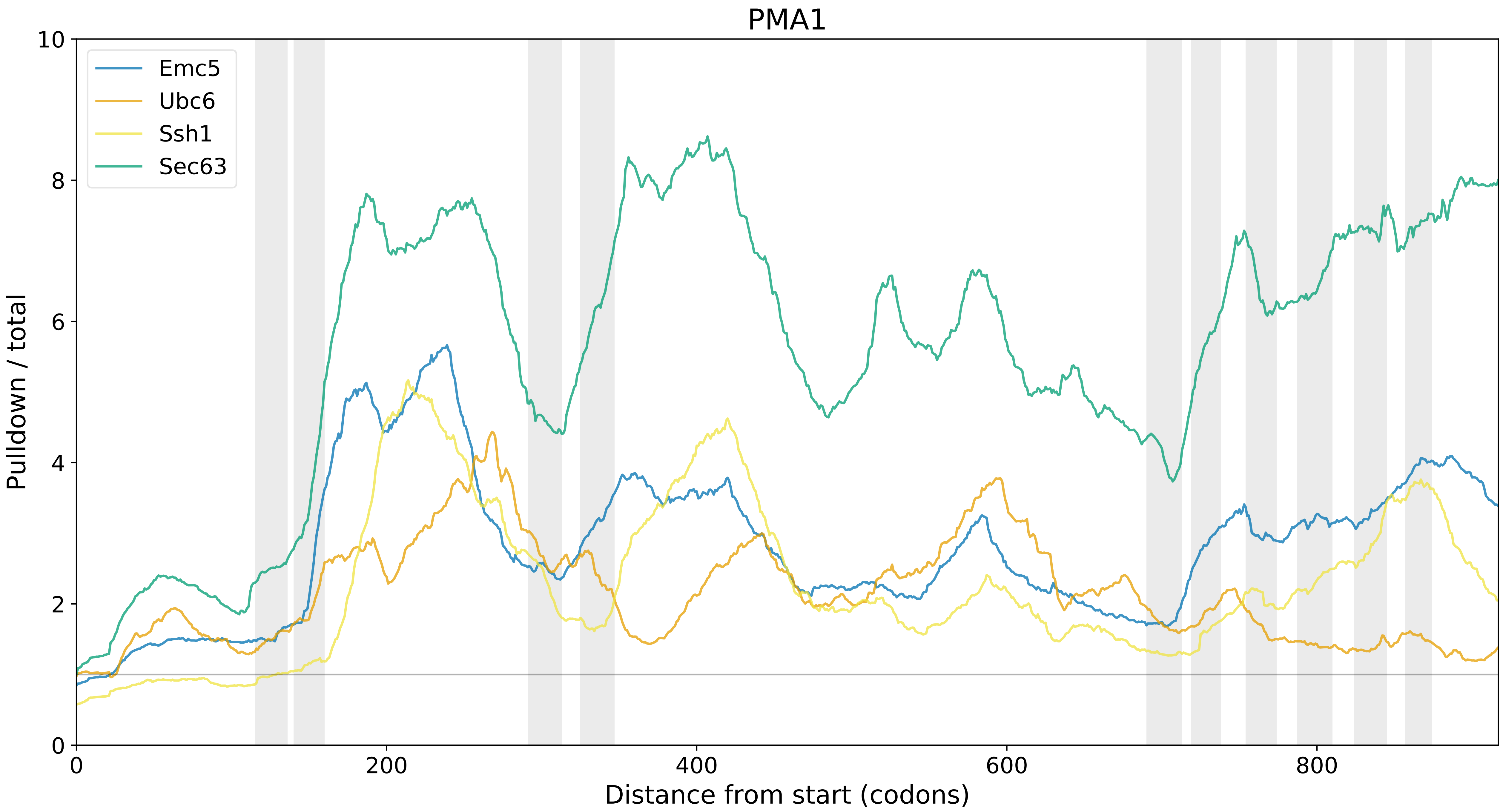

PMA1
Emc5
Ubc6
Ssh1
Sec63
Pulldown / total
Distance from start (codons)
0
2
4
6
8
10
0
200
400
600
800
